# Fully conjugated azacorannulene dimer as large diaza[80]fullerene fragment

**DOI:** 10.1038/s41467-022-29106-w

**Published:** 2022-03-21

**Authors:** Weifan Wang, Fiona Hanindita, Yosuke Hamamoto, Yongxin Li, Shingo Ito

**Affiliations:** Division of Chemistry and Biological Chemistry, School of Physical and Mathematical Sciences, Nanyang Technological University, 21 Nanyang Link, Singapore, 637371 Singapore

**Keywords:** Electronic properties and materials, Organic molecules in materials science, Synthetic chemistry methodology

## Abstract

A fully conjugated azacorannulene dimer with a large π-surface (76π system) was successfully synthesized from a fully conjugated bifunctional polycyclic aromatic azomethine ylide. This molecule represents an example of diaza[80]fullerene (C_78_N_2_) fragment molecule bearing two internal nitrogen atoms. X-ray crystallography analysis shows its boat-shaped structure with two terminal azacorannulenes bent in the same direction. The molecular shape leads to unique selective association with a dumbbell-shaped C_60_ dimer (C_120_) over C_60_ through shape recognition. Owing to its large π-surface and a narrow HOMO–LUMO gap, the azacorannulene dimer exhibits red fluorescence with a quantum yield of up to 31%. The utilization of the fully conjugated bifunctional azomethine ylide is a powerful method for the bottom-up synthesis of large multiazafullerene fragments, providing a step towards the selective total synthesis of multiazafullerenes.

## Abstract (web summary)

Heterofullerenes are an important synthetic target for organic chemists. Here, the authors demonstrate a fully conjugated azacorannulene dimer starting from a bifunctional polycyclic aromatic azomethine ylide, providing a powerful method for the bottom-up synthesis of large multiazafullerene fragments

## Introduction

Heterofullerene is a class of fullerenes in which one or more of its carbon atoms are substituted by heteroatoms such as nitrogen, boron, and phosphorous^1–4^. Since the substitution of carbon atoms within fullerene frameworks by heteroatoms is a feasible way to adjust its electronic and chemical properties, heterofullerenes are expected to find numerous potential applications in superconductors, optoelectronics, and organic semiconductors^5–7^. As such, heterofullerenes have been an important synthetic target for organic chemists. In contrast to the established methods of synthesizing fullerenes^8,9^, the synthetic process of heterofullerenes has long been a challenge^10^. The only successfully synthesized and isolated heterofullerene is an azafullerene, which contains nitrogen atoms within its framework. In 1995, Wudl et al. reported the first synthesis of azafullerene C_59_N in its dimeric form^11^. However, thus far, no multiazafullerene has been successfully synthesized and isolated on a macroscopic scale^12^, presumably due to its “isomeric problem”^13–15^. For instance, attempts to synthesize diazafullerene C_58_N_2_^16,17^ leads to the generation of 23 possible isomers^18–20^. Hence, currently the synthesis and isolation of a single isomer is still an open challenge.

**Fig. 1:**
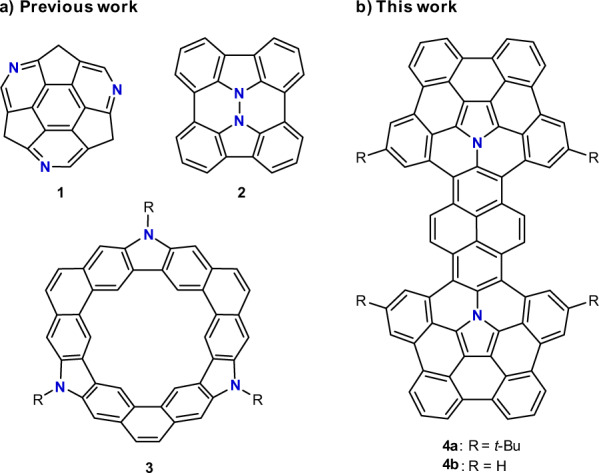
Representative multiazafullerene fragment molecules. **a** Molecules reported in literature. **b** Molecules reported in this manuscript.

Encouraged by the success for the “bottom-up” synthesis of C_60_ from well-designed aromatic precursors^21^, researchers have sparked off immense interest amongst the “bottom-up” synthesis of multiazafullerenes from azafullerene precursors. This synthetic approach will allow for a controlled and selective introduction of nitrogen atoms into fullerenes^22–25^. However, appropriate synthetic protocols to synthesize nitrogen-embedded polycyclic aromatic molecules as large azafullerene fragments are still lacking^26–29^. As far as we are aware, only a handful of multiazafullerene fragments have been reported^30–34^. As shown in Fig. 1a, triazasumanene **1**^30^ and “hydrazinobuckybowl” **2**^32^ are considered to be partial fragments of C_60-x_N_x_. Meanwhile, the molecular fragments of higher multiazafullerenes are scarce. To our knowledge, the examples include chrysaorole **3**^31^ and a corannulene molecule fused with two π-extended pyrroles^35^. It is worth noting that a pyrrolo[3,2-*b*]pyrrole-cored nanographene^36,37^ may be a good precursor for the synthesis of “isomeric multiazafullerenes”, which contain heptagon as well as pentagon and hexagon. In this regard, multiazafullerene fragment molecules are an attractive target, given the fact that the synthesis of multiazafullerene C_80-x_N_x_ (x ≥ 2) has not been achieved. During our continuous investigations on the synthesis of azafullerene fragment molecules^38–41^, we succeeded in achieving the bottom-up synthesis of diaza[80]fullerene fragment *t*-Bu_4_C_72_H_24_N_2_ (**4a** in Fig. 1b). This polycyclic aromatic molecule provides the largest π-surface of a [80]fullerene fragment bearing multiple heteroatoms.

## Results and discussion

### Synthesis and characterization

**Fig. 2:**
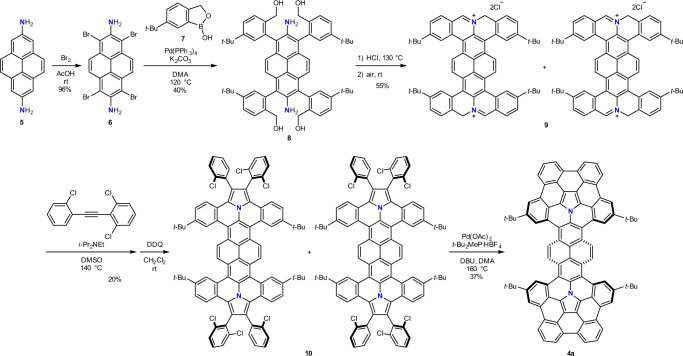
Synthetic route to azacorannulene dimer **4a**.

Our synthetic strategy shown in Fig. 2 started with the bromination of 2,7-diaminopyrene **5**^42,43^ to afford 1,3,6,8-tetrabromopyrene-2,7-diamine (**6**) in 96% yield. Subsequently, a palladium-catalyzed Suzuki-Miyaura cross-coupling reaction of **6** with an arylboronic acid **7** afforded the corresponding tetraarylated compound **8** in 40% yield. Afterward, an intramolecular cyclization of **8** by treatment with hydrogen chloride followed by air oxidation generates bifunctional iminium salt **9** in 55% yield as a mixture of regioisomers. Following the successful synthesis of iminium salt **9**, 1,3-dipolar cycloaddition with 2,2’,6-trichlorodiphenylethyne followed by oxidation with DDQ under ambient air was performed to generate fused pyrrole **10** in 20% yield. Finally, an intramolecular cyclization of **10** was carried out in the presence of Pd(OAc)_2_, (*t*-Bu)_2_MeP ∙ HBF_4_, and DBU^39^ to obtain **4a** in 37% yield. It is worth noting that **4a** should be stored under inert atmosphere due to its sensitivity to oxygen in a solution state, which is comparable to the corannulene/azacorannulene hybrid molecule in our previous report^38^. The structure of **4a** was confirmed by spectroscopic analysis. The ^1^H NMR spectrum exhibited three singlets, two doublets, and one triplet in aromatic region as well as one singlet in the aliphatic region, which are consistent with the *C*_2v_ symmetric structure of **4a**. In HRMS spectrum, an *m*/*z* value of 1145.4867, corresponding to an ion mass of C_88_H_61_N_2_ (*m*/*z* = 1145.4835), was observed as a major signal.

**Fig. 3:**
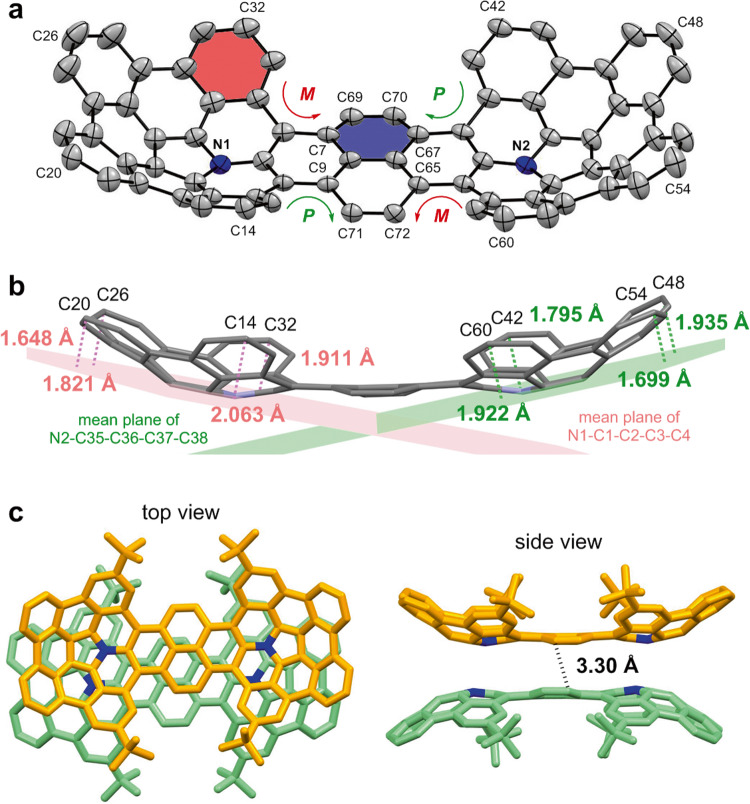
X-ray crystallographic analyses of azacorannulene dimer **4a**. **a** ORTEP structure of **4a** with thermal ellipsoids at 50% probability. Hydrogen atoms and *t*-butyl group are omitted. **b** Bowl depths of **4a**. Hydrogen atoms and *t*-butyl group are omitted. **c** Packing structure of **4a** in a unit cell. Hydrogen atoms are omitted.

The structure of **4a** was further confirmed by X-ray diffraction analysis of its single crystals, which were obtained by slow evaporation from its benzene/diethyl ether solution under argon atmosphere (Fig. 3). The ORTEP structure shows a boat-shaped structure with a fusion of two bowl-shaped azapentabenzocorannulene (APBC) moieties linked by a naphthalene unit. The two terminal azacorannulene bowls are bent in a syn-conformation. The central pyrene unit bends to give quadruple [4]helicene structures, in which two helicenes have a screw sense of *P* while the other two have *M* (Fig. 3a). The average interplanar angle between two terminal benzene rings (shown red and blue) of four [4]helicene units was determined to be 33.4°. This angle is larger than a substituted [4]helicene (25.1°)^44^ but is smaller than a hexabenzocoronene (42.5°)^45^. Due to the helicene structure, the central pyrene moiety is no longer planar. The dihedral angle between planes formed by C7-C69-C70-C67 and C9-C71-C72-C65 was determined to be 20.5°. The bowl depths, defined as the average perpendicular distance from the mean planes of the hub pyrrole rings (N1-C1-C2-C3-C4 and N2-C35-C36-C37-C38) to each summit atoms of C14, C32, C42 and C60, was determined to be 1.92 Å (Fig. 3b). The bowl depth is deeper than that of APBC (1.38–1.73 Å)^26^, which is attributed to the steric repulsion between hydrogen atoms in the [4]helicene structure. In the packing structure, two molecules of **4a** are packed as a dimeric form with a convex-to-convex π-π interaction (Fig. 3c). The shortest atomic distance between the two molecules is 3.30 Å, which is similar to that of a pentagon- and heptagon-embedded azabuckybowl (3.27 Å)^37^ and that of a pentagon- and heptagon-embedded nanographene (3.28 Å)^46^. These results indicate the presence of a strong intermolecular interaction.

### Conformational analysis

**Fig. 4:**
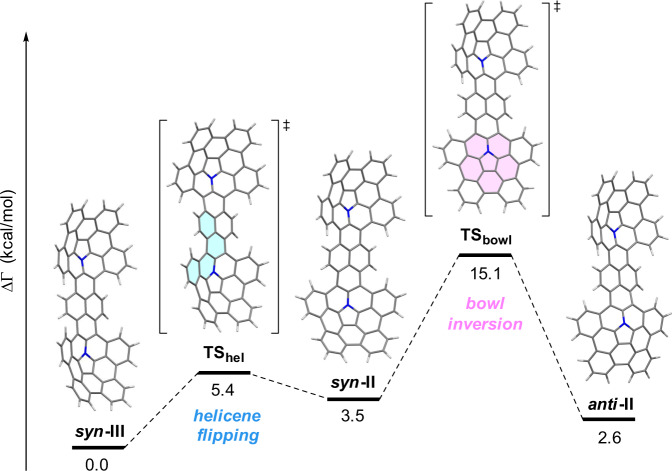
Interconversion pathways of **4b** calculated at the B3LYP/6-31G(d) level of theory. Blue highlights indicate a transition state that involves a helicene flipping of the [4]helicene moiety, while pink highlights indicate a transition state that involves a bowl inversion of the azacorannulene unit.

The conformation of molecule **4b**, in which *t*-butyl groups of **4a** are replaced by hydro groups, was analyzed by density functional theory (DFT) calculations at the B3LYP/6-31G(d) level of theory. Overall, there are 10 possible conformational isomers which are formed by combinations of the direction of two bowls (*syn*/*anti*) and the helicity of four [4]helicene units (*P*/*M*), whilst disregarding all enantiomers. Optimization starting from all the ten possible conformers resulted in eight local minimums. The Gibbs free energy values (kcal mol^−1^) relative to the most stable conformer are summarized in Supplementary Fig. 26. The most stable conformer was found to be ***syn*-III** (Fig. 4), which is in agreement with that observed in the X-ray diffraction analysis (Fig. 3) and is opposite to that of a similar bisdibenzocorannulene^47^. The relative energies of the other 7 conformers range within 5.6 kcal/mol. The transition states of some interconversions were also calculated. The conversion of ***syn*-III** into ***syn*-II**, which corresponds to the helicene flipping of one [4]helicene unit, has an activation energy of 5.4 kcal/mol. The energy of a bowl inversion (***syn*-III** to ***anti*-II**) was calculated to be 15.1 kcal/mol. Considering the reasonably low activation barriers for these interconversions, all the 8 conformers which gave local minimums can equilibrate at room temperature in solution state.

### Molecular properties

**Fig. 5:**
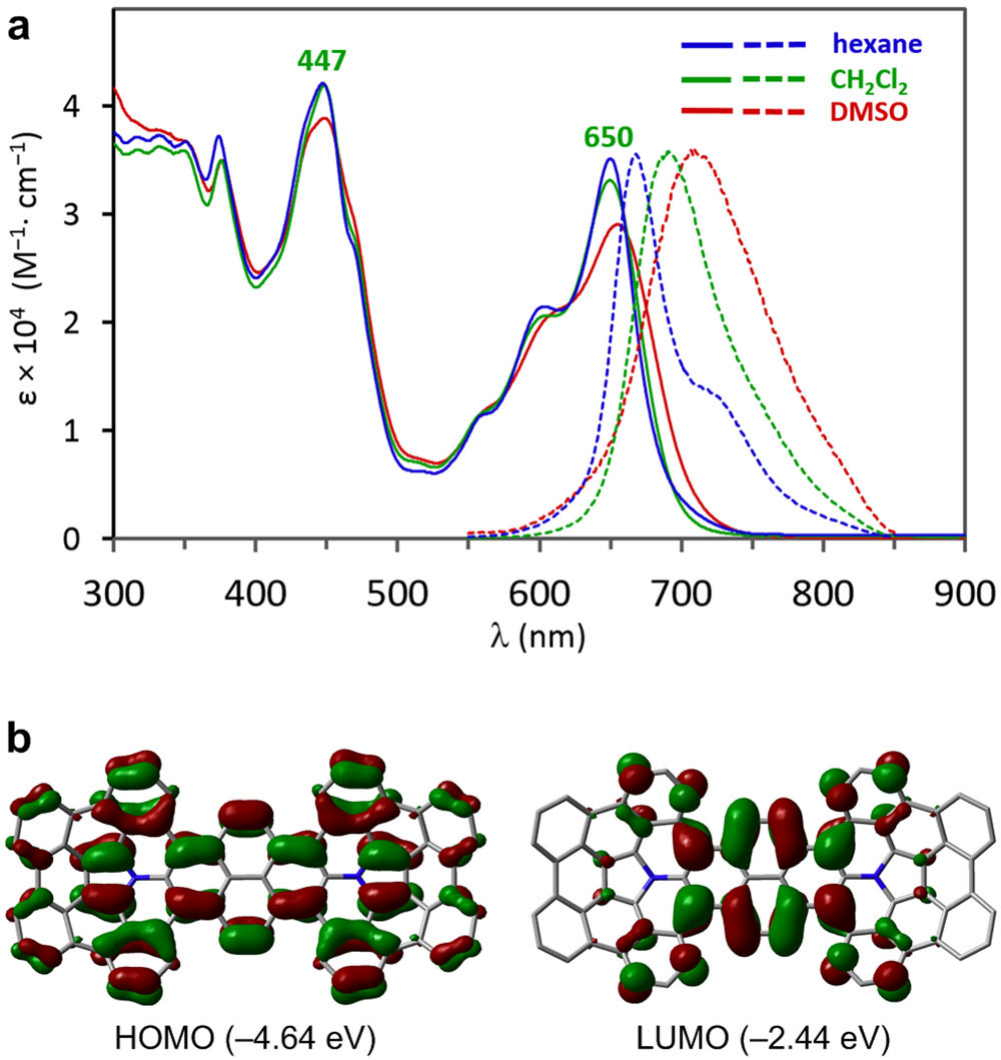
Optical Properties of azacorannulene dimer **4**. **a** UV/Vis absorption spectra (1.0 × 10^−6^ M, solid lines) and emission spectra (1.0 × 10^−5^ M, dashed lines) of **4a** in hexane (blue), dichloromethane (green), and dimethyl sulfoxide (red). **b** HOMO and LUMO of **4b**.

The optical properties of **4a** were assessed by absorption and emission spectroscopy (Fig. 5a). The green solution of **4a** in hexane, dichloromethane, and dimethyl sulfoxide exhibit comparable absorption bands at 300–720 nm. In dichloromethane, for instance, two major absorption peaks were observed at 447 and 650 nm, which are much larger than those of the parent APBC^26^ due to its extended π-conjugation system. A time-dependent DFT (TD-DFT) computation at the B3LYP/6-31G(d) level (Supplementary Table 4) indicates that the strong absorption band at around 650 nm corresponds to a HOMO-LUMO transition. To determine the HOMO-LUMO gap experimentally, the electrochemical properties of **4a** were investigated by cyclic voltammetry (CV) measurements (Supplementary Fig. 25). Compound **4a** showed two overlapped quasi-reversible oxidation peaks at *E* = *ca*. +0.16 and +0.25 V (vs. Fc/Fc^+^), while it showed one reversible reduction peak at *E* = − 1.77 V. The experimental HOMO-LUMO gap of **4a** was determined to be 1.94 eV, which is highly consistent with the absorption at 650 nm. Moreover, **4a** exhibits red fluorescence and indicates a positive solvatofluorochromic effect with maximum emission wavelengths (*λ*_em_) at 665 nm (hexane), 692 nm (dichloromethane), and 706 nm (dimethyl sulfoxide) with quantum yields of Φ_F_ = 0.31, 0.22, and 0.15, respectively. Since APBC shows a comparable fluorescence quantum yield of Φ_F_ = 0.24 in dichloromethane^26^, the extended π-conjugation of **4a** does not significantly affect its fluorescence quantum yield. The observed solvatofluorochromic phenomenon would be induced by the presence of intramolecular charge transfer due to donor-acceptor-donor nature of the molecule. Based on the fact that the HOMO is distributed all over the molecule including the two pyrrole moieties and that LUMO is mainly delocalized at the central pyrene moieties (Fig. 5b), the system involves the APBC cores as a donor moiety and the pyrene unit as an acceptor moiety. The lower fluorescence quantum yields in more polar solvents (Supplementary Table 2) as well as the redox properties in cyclic voltammetry also support our rationale^48^.

**Fig. 6:**
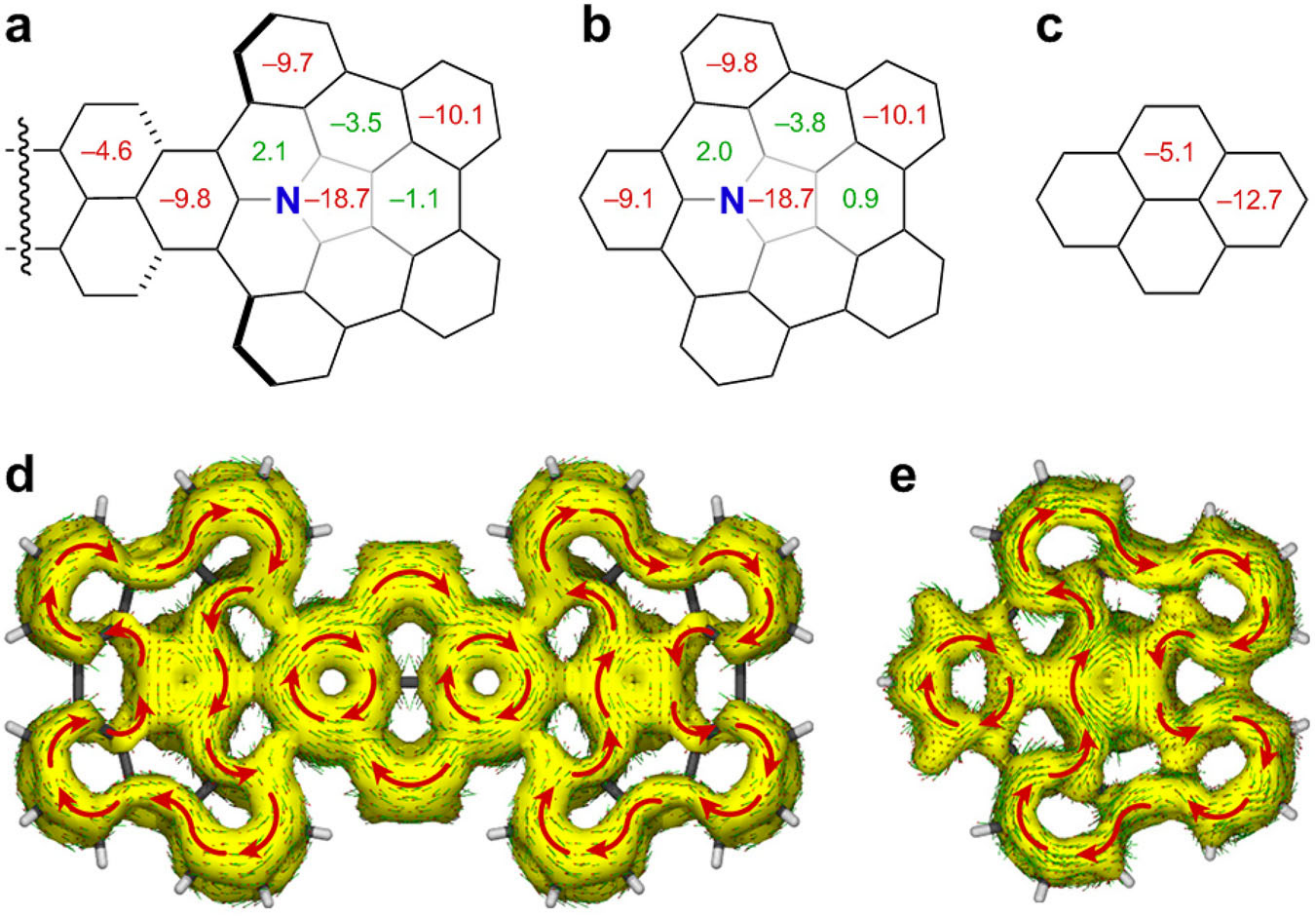
Aromatic properties of azacorannulene dimer **4b**. **a** NICS(0) values of **4b** calculated at the B3LYP/6-31G(d) level of theory. **b** NICS(0) values of APBC. **c** NICS(0) values of pyrene. **d** ACID plot of **4b**. **e** ACID plot of APBC.

The aromaticity of **4b** was characterized by nucleus independent chemical shift (NICS) analysis using DFT calculation at the B3LYP/6-31G(d) level of theory (Fig. 6). The large negative NICS values for the inner pyrrole core (−18.7 ppm) and its four outer benzene rings (−10.1 to −9.7 ppm) show that they are aromatic (Fig. 6a), which is in accordance with those of the reported APBC (Fig. 6b; −18.7 and −10.1 to −9.8 ppm)^26^. In addition, the central pyrene fragment in **4b** has comparable NICS values (−9.8 and −4.6 ppm) with pyrene (Fig. 6c; −12.7 and −5.1 ppm). The anisotropy of the induced current density (ACID) plot of **4b** in Fig. 6d shows that the central pyrene moiety has typical ring currents, in which two 6π benzene rings are connected by two carbon–carbon double bonds. In the azacorannulene moiety, clockwise (diamagnetic) 26π ring currents flowing along the core pyrrole moiety and the four outer benzene rings were observed, which substantiates the aromaticity by NICS calculation. These results show that the fusion of two APBC moieties does not significantly change their aromaticity.

### Host-guest chemistry

**Fig. 7:**
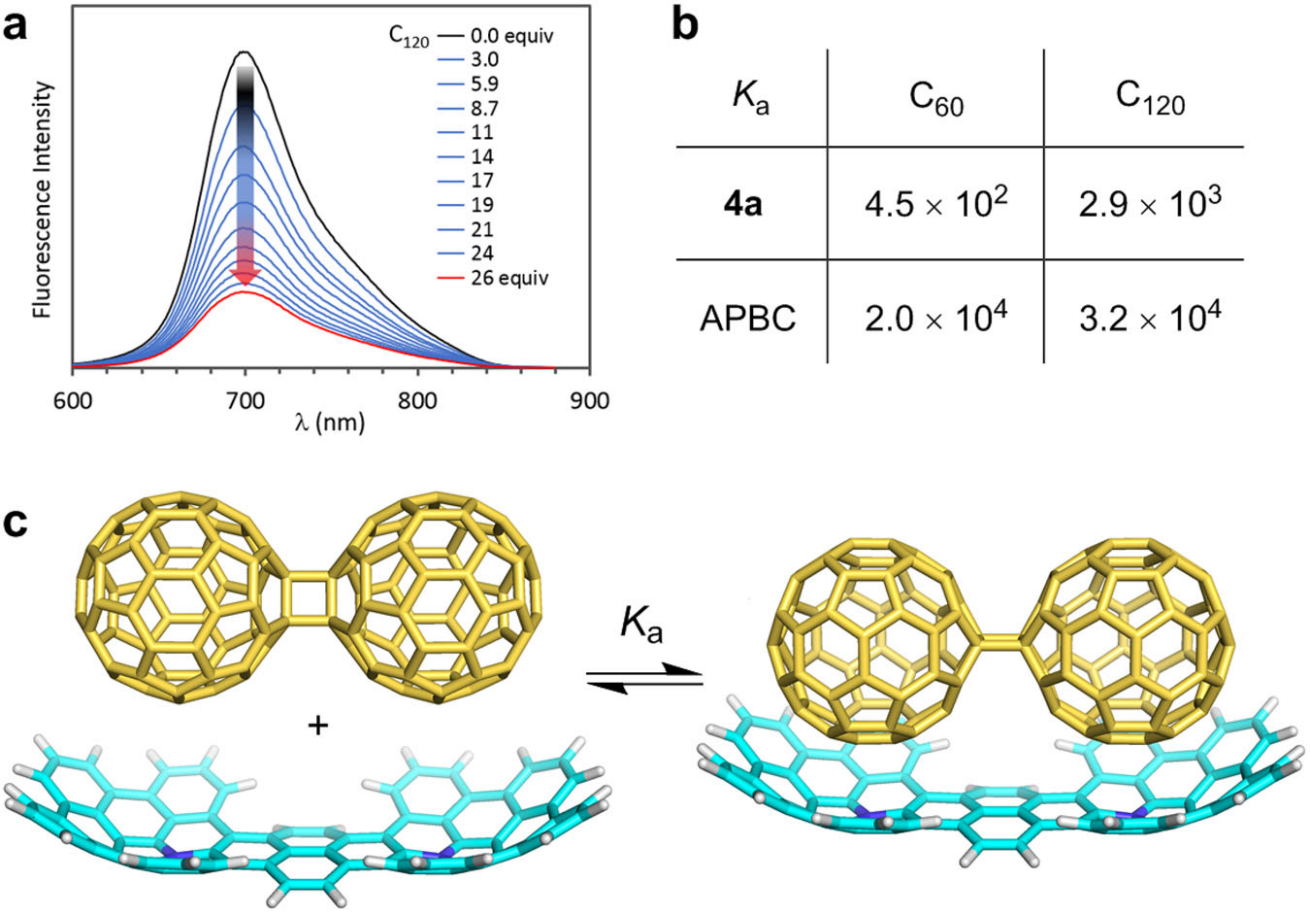
Host–guest chemistry between **4a** and C_120_. **a** Fluorescence spectra of **4a** upon titration with C_120_. **b** Association constants of host molecules (**4a** and APBC) and guest molecules (C_60_ and C_120_) determined by fluorescence titration. **c** One of the possible association modes of **4a** and C_120_ determined by DFT calculation at the B3LYP/6-31G(d) level with Grimme’s D3 dispersion correction.

During the investigation on the application of **4a**, we discovered its interesting shape-recognition behavior in host-guest chemistry. Inspired by the previous reports of azabuckybowls being utilized as buckycatchers^49,50^, association behavior of **4a** with C_60_ and a dumbbell-shaped C_60_ dimer (C_120_)^51^ was examined by fluorescence titration. As shown in Fig. 7a, the addition of C_120_ into a diluted solution of **4a** in 1,2-dichlorobenzene resulted in the gradual decrease of its fluorescence intensity. Based on the Benesi–Hildebrand equation, the association constants of **4a** toward C_60_ and C_120_ were determined to be *K*_a_(C_60_) = 4.5 × 10^2^ M^−1^ and *K*_a_(C_120_) = 2.9 × 10^3^ M^−1^ respectively (Fig. 7b), which indicate that **4a** favors C_120_ over C_60_ by one order of magnitude. The Job’s plot for the emission intensity indicates the formation of 1:1 supramolecular assembly of **4a** and C_120_ (Supplementary Fig. 21). It is worth noting that comparable association constants of APBC with C_60_ and C_120_ were observed (Fig. 7b), thus showing no distinct selectivity. These results strongly indicate that the boat-shaped structure of **4a** recognizes the dumbbell-shape of C_120_ during the association process in solution, leading to the higher association constant for C_120_ (Fig. 7c).

In summary, we have demonstrated the bottom-up synthesis of a diaza[80]fullerene fragment molecule **4a**. The large nitrogen-containing polycyclic aromatic molecule has a boat-shaped structure which can be viewed as the fusion of two bowl-shaped APBC moieties linked by a fused naphthalene unit. Conformational studies showed that a butterfly-butterfly conformer where the two azabuckybowls bend in the same direction is the most stable, which is consistent with that observed in X-ray diffraction analysis. The unique molecular shape leads to preferable association with a dumbbell-shaped C_60_ dimer (C_120_) over C_60_ through shape recognition. Theoretical analysis revealed the presence of a narrow HOMO-LUMO band gap, resulting in a strong absorption band at around 650 nm. The optical measurement exhibits a red fluorescence and solvatofluorochromic behavior. Importantly, the utilization of fully conjugated bifunctional polycyclic aromatic azomethine ylide **9** in a bottom-up synthetic approach provides a practical method for the selective synthesis of large multiazafullerene fragments.

## Methods

### Experimental procedure

The synthesis of **4a** is as follows: to a mixture of **10** (10 mg, 7.3 μmol), palladium diacetate (4.9 mg, 22 μmol) and di-*t*-butyl(methyl)phosphonium tetrafluoroborate (16 mg, 66 μmol) were added 1,8-diazabicyclo[5.4.0]undec-7-ene (DBU; 0.5 ml) and *N*,*N*-dimethylacetamide (DMA; 2.0 ml). The mixture was stirred for 19 h at 160 °C. After cooling to room temperature and dilution with toluene (5 ml), the mixture was washed with water (3 × 5 ml), dried over sodium sulfate, filtered, and concentrated in vacuo. The resulting mixture was purified by silica gel column chromatography with hexane/dichloromethane (4/1) to obtain **4a** as a dark green solid (3.1 mg, 2.7 μmol, 37%). Full experiment details can be found in the Supplementary Information.

### Theoretical calculations

All calculations were performed by using Gaussian 16 (revision A.03) program^52^ by the B3LYP method^53,54^ with the 6-31G(d) basis set^55,56^ for structure optimization, vibrational frequency, time-dependent density functional theory, NICS, and ACID calculations. Grimme’s D3 dispersion correction^57^ was used to investigate the association of **4b** with C_120_. Molecular geometries and transition state (TS) structures were optimized without any symmetry assumptions. Intrinsic reaction coordinate calculations were also performed for all TSs to ensure their true nature. All thermodynamics were obtained by utilizing the standard conditions at 298 K and 1 atm. Energies are presented as ΔG in kcal/mol.

## Supplementary information

Supplementary Information

## Data Availability

The data supporting the findings of the current study are available within the paper and its Supplementary Information or from the corresponding author upon request. The crystallographic data for compound **4a** have been deposited with the Cambridge Crystallographic Data Centre under deposition number 2103521 [https://www.ccdc.cam.ac.uk/solutions/csd-core/components/csd/].
