# Publisher Correction: Fully conjugated azacorannulene dimer as large diaza[80]fullerene fragment

**DOI:** 10.1038/s41467-022-29706-6

**Published:** 2022-04-05

**Authors:** Weifan Wang, Fiona Hanindita, Yosuke Hamamoto, Yongxin Li, Shingo Ito

**Affiliations:** Division of Chemistry and Biological Chemistry, School of Physical and Mathematical Sciences, Nanyang Technological University, 21 Nanyang Link, Singapore, 637371 Singapore

**Keywords:** Electronic properties and materials, Organic molecules in materials science, Synthetic chemistry methodology

Correction to: *Nature Communications* 10.1038/s41467-022-29106-w, published online 21 March 2022.

In this article references 14, 17, 25 and 32 were incorrect and should have been:

[14] Chen, Z., Reuther, U., Hirsch, A. & Thiel, W. Theoretical studies on the substitution patterns in heterofullerenes C_70-x_N_x_ and C_70-x_B_x_ (x = 2–10). *J. Phys. Chem. A* **105**, 8105–8110 (2001).

[17] Huang, H. et al. Synthesis of an azahomoazafullerene C_59_N(NH)R and gas-phase formation of the diazafullerene C_58_N_2_. *Angew. Chem. Int. Ed*. **52**, 5037–5040 (2013).

[25] Liu, J. & Feng, X. Bottom-up synthesis of nitrogen-doped polycyclic aromatic hydrocarbons. *Synlett* **31**, 211–222 (2020).

[32] Higashibayashi, S., Pandit, P., Haruki, R., Adachi, S.-i. & Kumai, R. Redox-dependent transformation of a hydrazinobuckybowl between curved and planar geometries. *Angew. Chem. Int. Ed*. **55**, 10830–10834 (2016).

The original article has been corrected.

