# Supplementary material for: Fully conjugated azacorannulene dimer as large diaza[80]fullerene fragment
Source: Nat Commun. 2022 Mar 21;13:1498. doi: 10.1038/s41467-022-29106-w (PMC8938435; doi:10.1038/s41467-022-29106-w)
Supplement: Supplementary file 1 — Supplementary Information [file 41467_2022_29106_MOESM1_ESM.pdf]

Supplementary Information for:

Fully Conjugated Azacorannulene Dimer as Large Diaza[80]fullerene Fragment

Weifan Wang, Fiona Hanindita, Yosuke Hamamoto, Yongxin Li, Shingo Ito*

Division of Chemistry and Biological Chemistry, School of Physical and Mathematical Sciences, Nanyang Technological University, Singapore 637371, Singapore

Table of Contents

1. General	2
2. Synthesis of Compounds	3–7
3. NMR Spectra	8–15
4. X-Ray Crystallographic Data	16–17
5. Optical Properties	18
6. Association with Fullerenes	20
7. Electrochemical Properties	22
8. Theoretical Calculations	23–42
9. References	43

1. General

General: All reactions were carried out in a glove box or using standard Schlenk techniques under argon or nitrogen atmosphere unless otherwise noted. Thin-layer chromatography (TLC) was performed using glass plates pre-coated with silica gel impregnated with a fluorescent indicator (Merck, #1.15685.0001). Silica gel column chromatography was performed as described by Still, et al.,[1] employing silica gel (Davisil, 60 Å, 40-63 micron) purchased from Sigma-Aldrich.

Instrumentation: Nuclear magnetic resonance (NMR) spectra were recorded on Bruker AV 500 (^{1}H: 500 MHz and ^{13}C: 126 MHz), BBFO (^{1}H: 400 MHz and ^{13}C: 101 MHz), AV 800 (^{1}H: 800 MHz and ^{13}C: 201 MHz) NMR spectrometers. Chemical shift values for ^{1}H are referenced to tetramethylsilane (δ 0.00 ppm) or the residual signal of chloroform-*d* (δ 7.26), methylene chloride-d_2 (δ 5.32), methanol-d_4 (δ 3.31) or benezene-d_6 (δ 7.16) and chemical shift values for ^{13}C are referenced to the carbon resonance of chloroform-*d* (δ 77.2), methylene chloride-d_2 (δ 54.0), methanol-d_4 (δ 49.0) or C_6D_6 (δ 128.1). Preparative HPLC separation was carried out with a LaboACE LC-5060 (Japan Analytical Industry Co. Ltd.) equipped with a GPC column (Japan Analytical Industry Co. Ltd.; JAIGEL-2HR) by eluting with chloroform (8 mL/min) at room temperature. High-resolution mass (HRMS) spectra were taken on a Waters Q-Tof Premier mass spectrometer with an electron spray ionization time-of-flight (ESI-TOF) method or on a Bruker Autoflex III mass spectrometer with a matrix-assisted laser desorption/ionization time-of-flight (MALDI-TOF) method. Infrared (IR) spectra were recorded on a PerkinElmer FTIR spectrum-100 spectrometer with an attenuated total reflection (ATR) system. Ultraviolet/visible (UV/vis) absorption spectra were recorded on a Shimadzu UV-1800 spectrometer. Fluorescence spectra were recorded on JASCO FP-8500 Spectrofluorometer. Decomposition points and melting points were recorded on an OptiMelt MPA-100 apparatus. Cyclic voltammetry and differential pulse voltammetry were recorded on a BioLogic SP-50 electrochemical analyzer. X-ray crystallography analysis was performed on Bruker D8 APEX X-ray diffractionmeter.

Materials: The following reagents were purchased from the indicated suppliers and used as received: bromine (Alfa Aesar, 98%), 4-*tert*-butylbenzyl alcohol (Aldrich, 95%), butyllithium (Aldrich, 99%), *N*,*N*,*N*,*N*-tetramethylethylenediamine (Aldrich, 98%), trimethyl borate (Aldrich, 98%), tetrakis(triphenylphosphine)palladium (Aldrich, 99%), hydrogen chloride in 1,4-dioxane (Tokyo Chemical Industry Co., Ltd. (TCI), 99%), *N*,*N*-diisopropyl(ethyl)amine (TCI, 97%), 2,3-dichloro-5,6-dicyano-*p*-benzo-quinone (DDQ; TCI, 97%), palladium(II) acetate (Aldrich, 97%), [*t*-Bu_2MePH][BF_4] (Aldrich, 97%), 1,8-diazabicyclo[5.4.0]undec-7-ene (DBU, TCI, 98%), anhydrous dimethyl sulfoxide (DMSO; Aldrich, 99.9%), anhydrous *N*,*N*-dimethylacetamide (DMA; Aldrich, 99.8%). The following reagents were prepared according to the literature procedures: 2,7-diaminopyrene (**5**)[2] and 2,2',6-trichlorodiphenyl-ethyne.[3]

2. Synthesis of Compounds

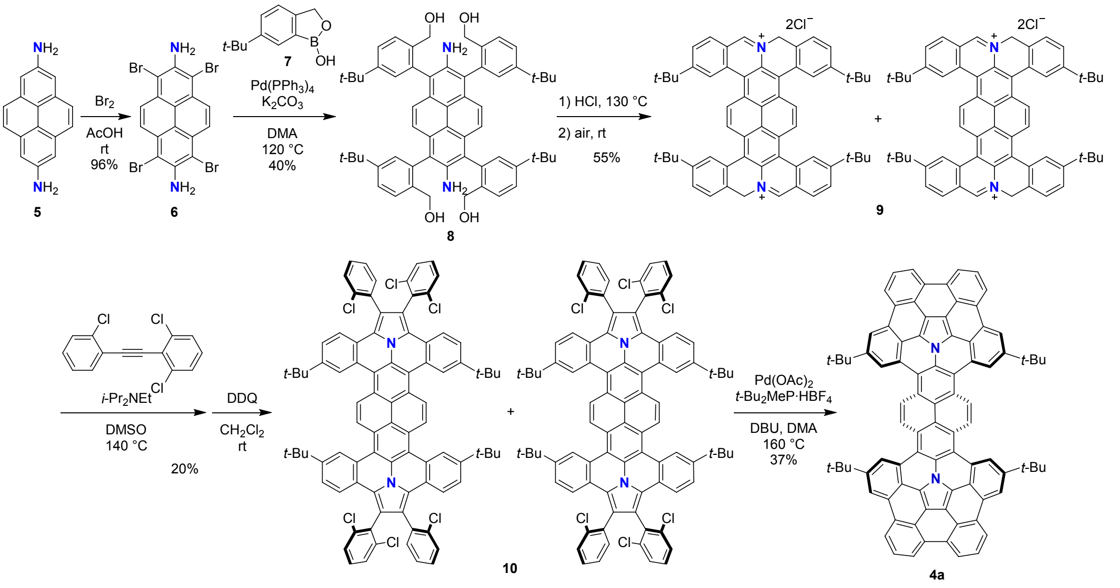

Supplementary Figure 1. Synthetic route to compound **4a**.

1,3,6,8-tetrabromopyrene-2,7-diamine (6)

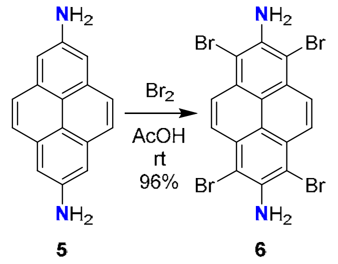

To a solution of **5** (0.20 g, 0.86 mmol) in acetic acid (20 mL) was added bromine (1.24 g, 7.9 mmol) dropwise and the mixture was stirred for 14 h at room temperature. The black precipitate was filtered and washed with ethanol (5×30 mL), compound **6** was obtained as a black solid (0.45 g, 0.83 mmol, 96%). mp > 300 °C (dec.); IR (neat); cm^{-1} 3414, 3315, 2158, 2034, 1910, 1711, 1596, 1447, 1406, 1307, 1166, 1091, 1034, 1000, 803. Although the obtained compound was insoluble in all common organic solvents, it was identified as **6** by MALDI-TOF-MS, MS (MALDI) *m/z* calcd for $C_{16}H_8N_2Br_4$ $[M]^+$ 543.7399, found 543.7378.

6-*tert*-butylbenzo[*c*][1,2]oxaborol-1(3*H*)-ol (7)

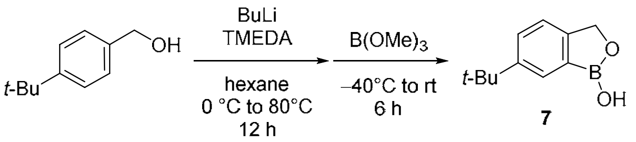

To a solution of 4-*tert*-butylbenzyl alcohol (9.0 g, 55 mmol), tetramethylethylenediamine (14.0 g, 120 mmol) in hexane (37 mL) was added butyllithium (2.0 M solution in hexane; 60 mL, 120 mmol) dropwise at 0 °C for 10 min. The mixture was stirred for 12 h under refluxed

conditions. Subsequently, the mixture was cooled down to −40 °C and trimethyl borate (19.9 g, 192 mmol) was added dropwise. The mixture was stirred for 6 h at room temperature. The reaction mixture was quenched with 15 wt% HCl (177 mL) at 0 °C and extracted with dichloromethane (3×100 mL). The combined organic phase was washed with 2N NaOH (3×100 mL). The aqueous phase was combined and acidified to pH of *ca.* 2 by 6 N HCl. The resulting white precipitate was extracted with dichloromethane (3×100 mL). The combined organic phase was washed with water, dried over sodium sulfate. After filtration and evaporation, the crude product was purified by silica gel column chromatography eluted with hexane/ethyl acetate (4/1) to obtain **7** as a white solid (3.11 g, 16.4 mmol, 30 %). R_f = 0.25 (hexane/ethyl acetate = 4/1); mp 105–107 °C; IR (neat) cm^{-1} 3292, 2950, 2909, 2876, 1614, 1500, 1467, 1435, 1394, 1361, 1296, 1255, 1214, 1117, 1068, 1035, 970, 873, 824, 783, 759, 734, 693; ^{1}H NMR (500 MHz, $CDCl_3$, 297 K) δ 7.80 (s, 1H), 7.56 (dd, *J* = 8.0, 2.0 Hz, 1H), 7.30 (d, *J* = 8.0 Hz, 1H), 5.42 (s, 1H), 5.10 (s, 2H), 1.36 (s, 9H); ^{13}C NMR (126 MHz, $CDCl_3$, 297 K) δ 151.0(2×1C), 150.4, 128.8, 127.1, 120.9, 71.3, 34.9, 31.7(3C); ^{11}B NMR (128 MHz, $CDCl_3$, 297 K) δ 33.1; HRMS (ESI) *m*/*z* calcd for $C_{11}H_{16}O_2B$ $[M+H]^+$ 191.1243, found 191.1251.

1,3,6,8-tetra[(2'-hydroxymethyl)-4'-(*tert*-butyl)phenyl]pyrene-2,7-diamine (8)

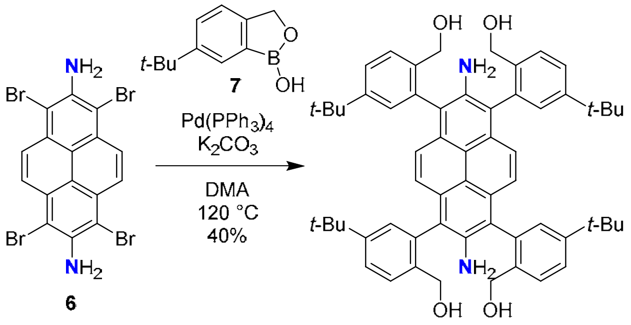

A solution of **6** (0.24 g, 0.44 mmol), arylboronic acid **7** (0.46 g, 2.4 mmol), $Pd(PPh_3)_4$ (0.45 g, 0.39 mmol) and potassium carbonate (1.1 g, 7.9 mmol) in DMA (30 mL) was stirred for 22 h at 120 °C. After cooling to room temperature, dilution with ethyl acetate (60 mL), the mixture was washed with water (3×30 mL), then dried over sodium sulfate. After filtration and evaporation, the crude product was purified by silica gel column chromatography eluted with hexane/ethyl acetate (3/2) to obtain **8** as a dark yellow solid (0.15 g, 0.17 mmol, 40%). R_f = 0.40 (hexane/ethyl acetate = 3/2); mp 270–272 °C; IR (neat) cm^{-1} 3576, 3382, 3334, 2957, 2869, 1768, 1601, 1552, 1464, 1383, 1351, 1287, 1215, 1094, 1006, 894, 830, 797, 749, 725, 669; ^{1}H NMR (500 MHz, $CDCl_3$, 297 K) δ 7.62 (d, *J* = 8.0 Hz, 4H), 7.54 (dd, *J* = 8.0, 2.0 Hz, 4H), 7.38 (d, *J* = 2.0 Hz, 4H), 7.34 (s, 4H), 4.35–4.30 (m, 8H), 3.63 (broad s, 4H), 2.64 (broad s, 4H), 1.34 (s, 36H); ^{13}C NMR (126 MHz, $CDCl_3$, 297 K) δ 152.2(4C), 139.0(2C), 137.7(4C), 135.3(4C), 129.6(4C), 129.0(4C), 128.4(4C), 126.1(4C), 125.3(4C), 123.5(4C), 119.9(2C), 63.8(4C), 34.9(4C), 31.6(12C); HRMS (ESI) *m*/*z* calcd for $C_{60}H_{69}N_2O_4$ $[M+H]^+$ 881.5257, found 881.5264.

3,8,15,20-Tetra-*t*-butyl-11a,23a-diazatribenzo[*a*,*o*,*rst*]dinaphtho[3,2,1-*de*:1',2',3'-*kl*]pentaphene (9)

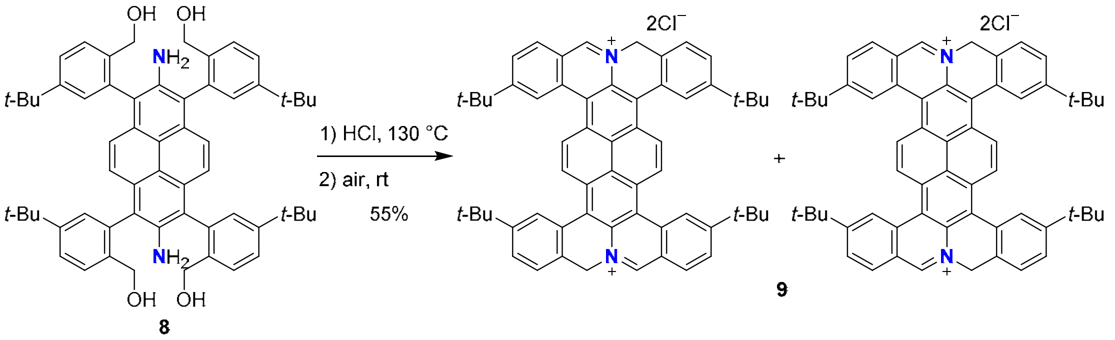

To a 25-mL Schlenk tube were added **8** (156 mg, 0.177 mmol) and hydrogen chloride (4.0 M solution in dioxane; 4.0 mL, 16 mmol) under argon atmosphere. The mixture was stirred for 20 h at 130 °C. After cooling to room temperature and the removal of the cap of the Schlenk tube, the reaction mixture was stirred in air for 20 h. After adding diethyl ether (20 mL), the formed precipitate was collected by filtration. The precipitate was washed with diethyl ether (3×10 mL) and hexane (3×10 mL) to obtain **9** as a dark red-brown solid (86 mg, 0.098 mmol, 55%). mp >300 °C (dec.); IR (neat) cm^{-1} 3355, 2959, 2868, 2157, 1975, 1604, 1496, 1471, 1405, 1355, 1256, 1190, 1157, 1107, 1033, 909, 835, 744; ^{1}H NMR (500 MHz, CD_3OD, 297 K) δ 10.42 (s, 2H), 9.76 (s, 2H), 9.72 (s, 2H), 9.13 (s, 2H), 8.84 (d, *J* = 8.5 Hz, 2H), 8.48 (d, *J* = 8.5 Hz, 2H), 8.35 (s, 2H), 7.87 (d, *J* = 8.0 Hz, 2H), 7.82 (d, *J* = 8.0 Hz, 2H), 6.29 (s, 4H), 1.77 (s, 18H), 1.52 (s, 18H); ^{13}C NMR (126 MHz, CD_3OD, 297 K) δ 164.7(2C), 154.2(2C), 154.2(2C), 135.8(2C), 134.1(2C), 132.5(2C), 130.7(2C), 130.3(2C), 130.3(2C), 130.0(2C), 129.2(2C), 129.0(2C), 129.0(2C), 128.9(2C), 128.7(2C), 128.3(2C), 127.5(2C), 125.7(2C), 125.6(2C), 125.4(2C), 123.3(2C), 60.3(2C), 38.1(2C), 36.2(2C), 31.8(6C), 31.4(6C); HRMS (ESI) *m*/*z* calcd for $C_{60}H_{58}N_2$ $[M-Cl_2]^{2+}$ 403.2300, found 403.2311.

3,8,15,20-tetra-*t*-butyl-11,23-bis(2-chlorophenyl)-12,24-bis(2,6-dichlorophenyl)-10b^1,22b^1-diazatribenzo[*a*,*o*,*rst*]benzo[4,5]indeno[1,7,6-*cde*]benzo[4,5]indeno[6,7,1-*klm*]pentaphene
3,8,15,20-tetra-*t*-butyl-11,24-bis(2-chlorophenyl)-12,23-bis(2,6-dichlorophenyl)-10b^1,22b^1-diazatribenzo[*a*,*o*,*rst*]benzo[4,5]indeno[1,7,6-*cde*]benzo[4,5]indeno[6,7,1-*klm*]pentaphene (10)

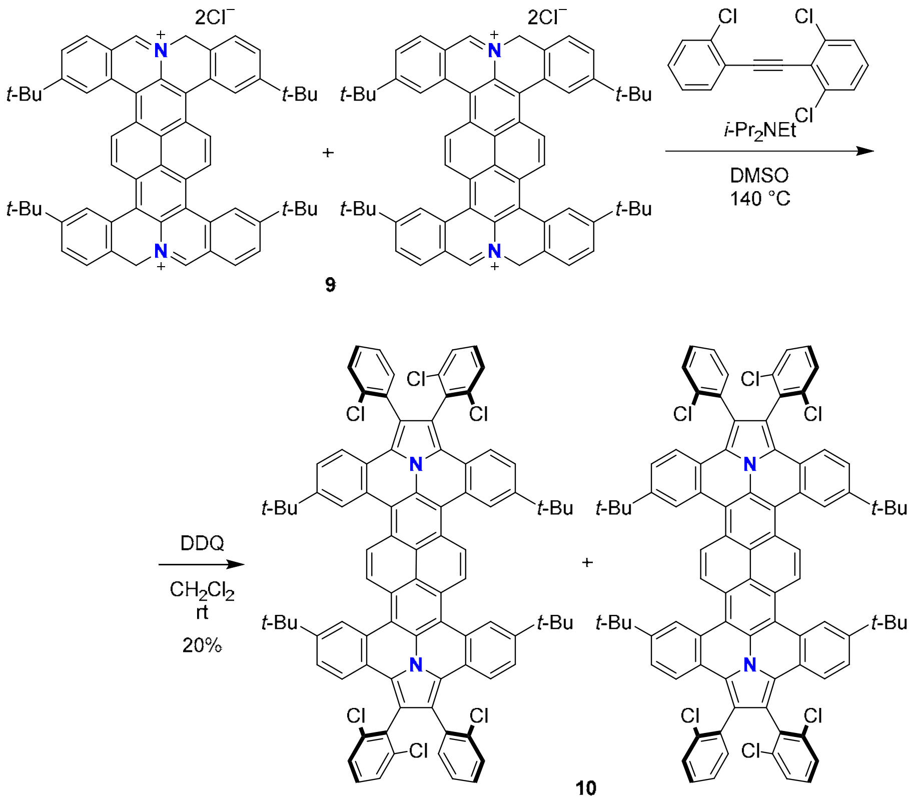

To a preheated solution of 1-(2-chlorophenyl)-2-(2,6-dichlorophenyl)ethyne (58 mg, 0.21 mmol) and *N*,*N*-diisopropyl(ethyl)amine (93 μL, 0.55 mmol) in DMSO (12 mL) was added **9** (60 mg, 0.068 mmol) at 140 °C. The mixture was stirred for 36 h at 140 °C. After cooling to room temperature and dilution with toluene (30 mL), the mixture was washed with water (3×30 mL) and evaporated *in vacuo*. The crude mixture was dissolved in dichloromethane (10 mL), and to the solution was added DDQ (28 mg, 0.12 mmol). After stirring for 15 min at room temperature, the reaction mixture was washed with a saturated aqueous solution of sodium bicarbonate (10 mL) and extracted with dichloromethane (3×10 mL). The organic phases were dried over sodium sulfate, filtrated, and evaporated *in vacuo*. The crude product was purified by silica gel column chromatography. The column was first eluted with hexane to remove recovered 1-(2-chlorophenyl)-2-(2,6-dichlorophenyl)ethyne and next eluted with hexane/dichloromethane (4/1) to obtain **10** as a red-blue solid (18.9 mg, 0.013 mmol, 20%). R_f = 0.25 (hexane/dichloromethane = 4/1); mp > 300 °C (dec.); IR (neat) cm^{-1} 3059, 2951, 2860, 2158, 2025, 1910, 1728, 1596, 1555, 1456, 1406, 1356, 1265, 1191, 1092, 1017, 908, 811, 778, 753, 678; ^{1}H NMR (500 MHz, CD_2Cl_2/CS_2, 297 K) δ 9.26–9.25 (m, 2H), 9.22–9.20 (m, 2H), 9.16–9.13 (m, 4H), 7.68 (d, *J* = 8.5 Hz, 1H), 7.61–7.54 (m, 5H), 7.52–7.49 (m, 5H), 7.46–7.33 (m, 9H), 7.30 (t, *J* = 7.5 Hz, 1H), 7.24 (t, *J* = 7.5 Hz, 1H), 1.60–1.58 (m, overlapping, 36H); ^{13}C NMR (201 MHz, CD_2Cl_2/CS_2, 298 K) δ 149.2, 149.0, 138.0, 137.5, 137.5, 137.1, 135.9,

135.7, 134.8, 134.7, 134.6, 133.1, 133.0, 132.4, 129.9, 129.8, 129.1, 128.9, 128.8, 128.7, 128.4, 128.4, 128.1, 126.7, 126.6, 126.5, 126.4, 126.3, 126.1, 126.1, 126.0, 125.6, 125.5, 125.2, 125.2, 125.2, 123.7, 122.9, 122.8, 122.5, 122.5, 122.0, 122.0, 121.8, 119.1, 118.9, 118.7, 118.6, 118.4, 116.7, 116.7, 35.2, 35.2, 31.7 (Note: Carbon numbers could not be assigned due to the complex signals resulting from a mixture of isomers); HRMS (ESI) *m/z* calcd for $C_{88}H_{67}N_2Cl_6$ $[M+H]^+$ 1363.3406, found 1363.3455.

2,11,16,25-tetra-*t*-butyl-4,76-diaza-21,40:22,43:34,55:35,36:41,42:56,57-hexaseco-24,28,32,45,49,53-hexanor(C_{80}-D_{5h})[5,6]fullerene (4a)

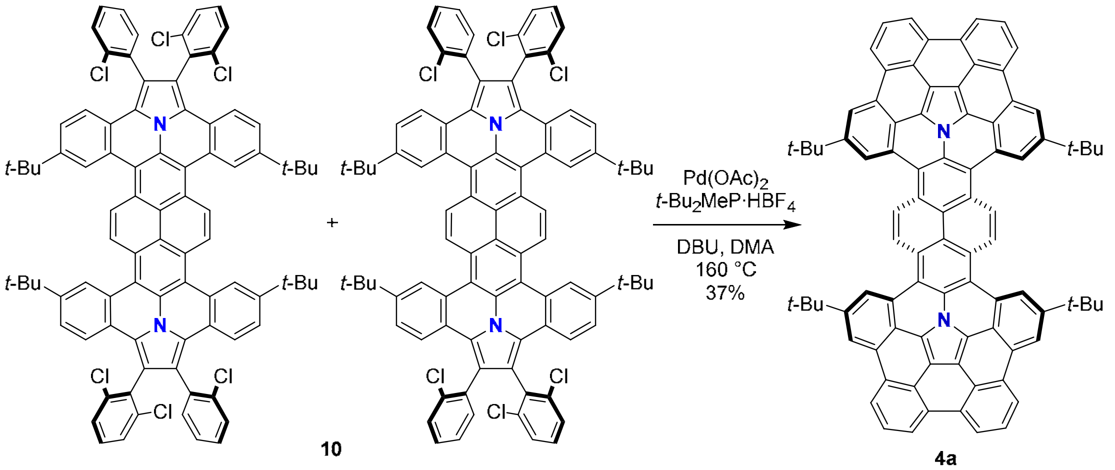

To a mixture of **10** (10 mg, 7.3 µmol), palladium diacetate (4.9 mg, 22 µmol) and di-*tert*-butyl(methyl)phosphonium tetrafluoroborate (16 mg, 66 µmol) in a 25-mL Schlenk tube were added DBU (0.5 mL) and DMA (2.0 mL) via a syringe. Nitrogen gas was bubbled to the solution for 10 min. The mixture was stirred for 19 h at 160 °C. After cooling to room temperature and dilution with toluene (5 mL), the mixture was washed with water (3×5 mL), then dried over sodium sulfate, filtrated, and evaporated *in vacuo*. The crude product was purified by silica gel column chromatography eluted with hexane/dichloromethane (4/1) to obtain **4a** as a dark green solid (3.1 mg, 2.7 µmol, 37%). (Note: Since **4a** was found to be slightly sensitive to oxygen in a solution state, after the purification, **4a** was immediately taken into a glove box and stored). R_f = 0.25 (hexane/dichloromethane = 4/1); mp > 300 °C (dec.); IR (neat) cm^{-1} 3059, 2951, 2918, 2852, 2167, 2009, 1719, 1628, 1538, 1505, 1455, 1406, 1356, 1257, 1158, 1042, 1009, 869, 818, 803, 736, 679; ^{1}H NMR (400 MHz, C_6D_6, 297 K) δ 9.94 (s, 4H), 9.48 (s, 4H), 8.87 (s, 4H), 8.57 (d, *J* = 8.0 Hz, 4H), 8.37 (d, *J* = 7.6 Hz, 4H), 7.69 (t, *J* = 8.0 Hz, 4H), 1.69 (s, 36H); ^{13}C NMR (201 MHz, C_6D_6/CS_2, 298 K) δ 149.1(4C), 137.4(4C), 133.8(4C), 133.4(4C), 132.7(4C), 131.2(4C), 130.8(4C), 129.7(4C), 129.3(4C), 126.0(4C), 125.7(4C), 124.8(4C), 124.5(4C), 123.8(4C), 122.9(4C), 122.8(4C), 122.3(4C), 120.2(4C) (one aromatic peak is overlapped with that of C_6D_6), 36.0(4C), 32.0(12C); ^{13}C NMR (201 MHz, $CDCl_3/CS_2$, 298 K) δ 149.3, 137.9, 133.8, 132.9, 132.2, 130.8, 130.3, 129.4, 129.3, 127.1, 125.7, 125.4, 124.9, 124.2, 124.0, 122.9, 122.8, 122.3, 120.0, 36.2, 32.2 (Note: Sharp NMR signals were observed only in non-polar solvents such as C_6D_6 or CS_2 probably due to aggregation in polar solvents); HRMS (ESI) *m/z* calcd for $C_{88}H_{61}N_2$ $[M+H]^+$ 1145.4835, found 1145.4867.

3. NMR Spectra

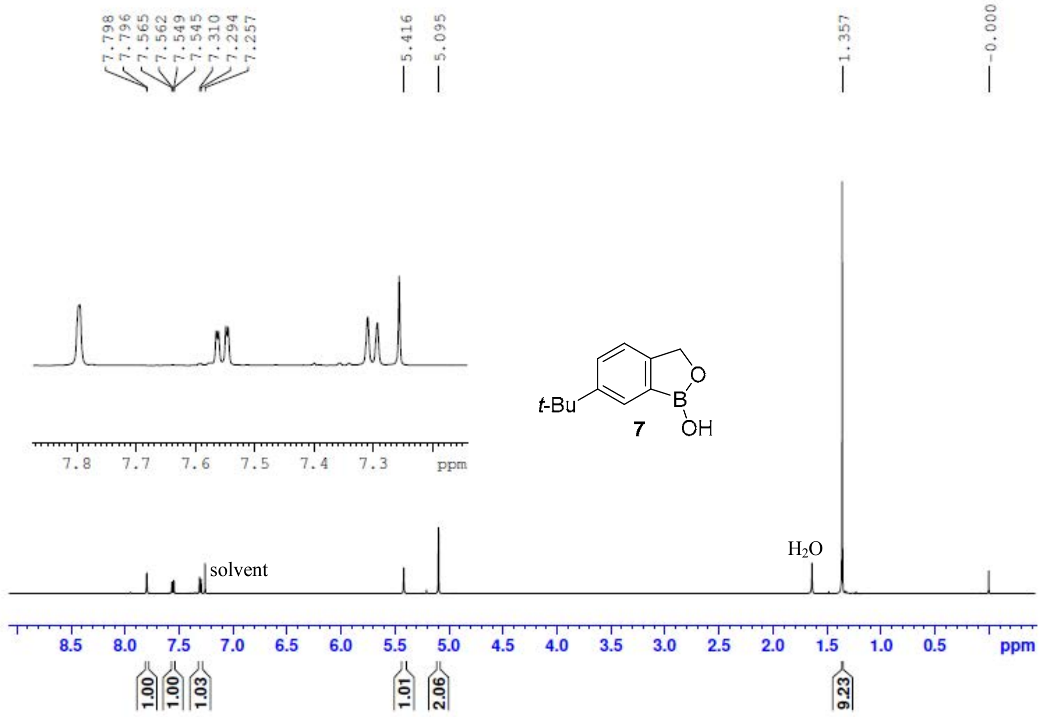

Supplementary Figure 2. ^{1}H NMR spectrum of **7** (500 MHz, $CDCl_3$, 297 K).

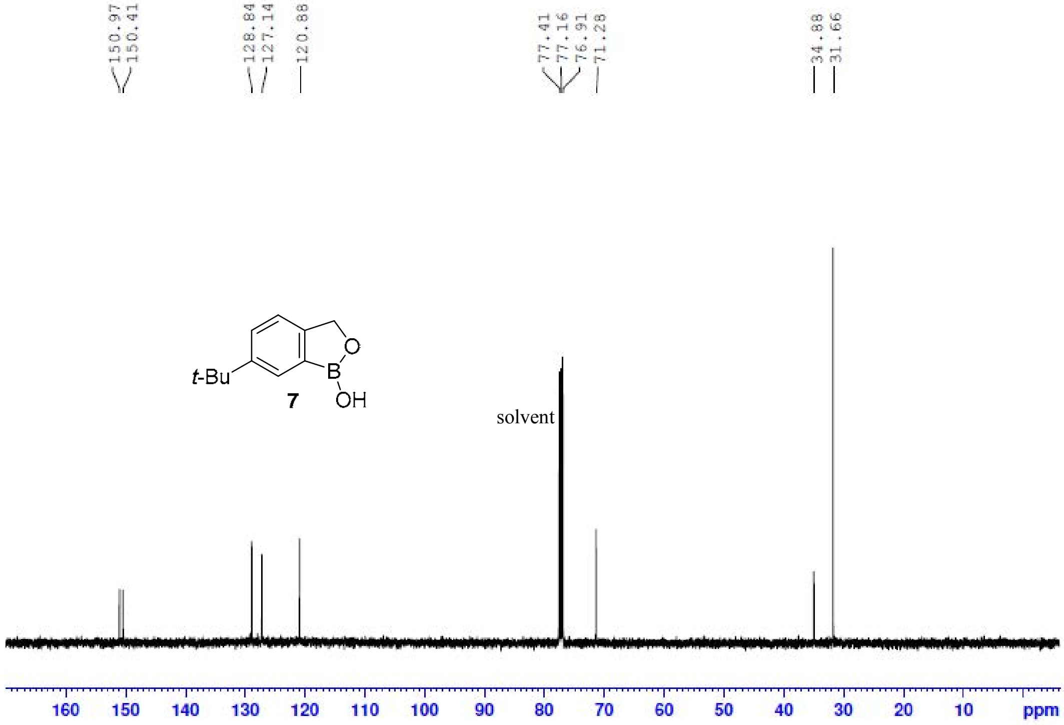

Supplementary Figure 3. ^{13}C NMR spectrum of **7** (126 MHz, $CDCl_3$, 297 K).

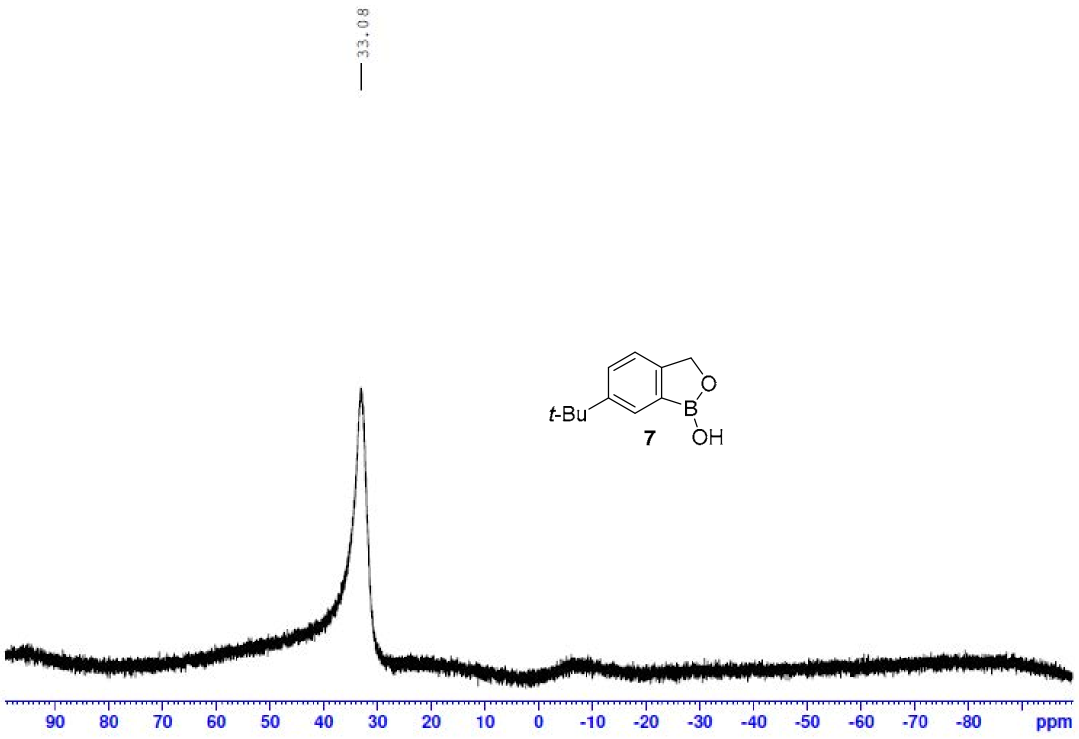

Supplementary Figure 4. ^{11}B NMR spectrum of **7** (128 MHz, $CDCl_3$, 297 K).

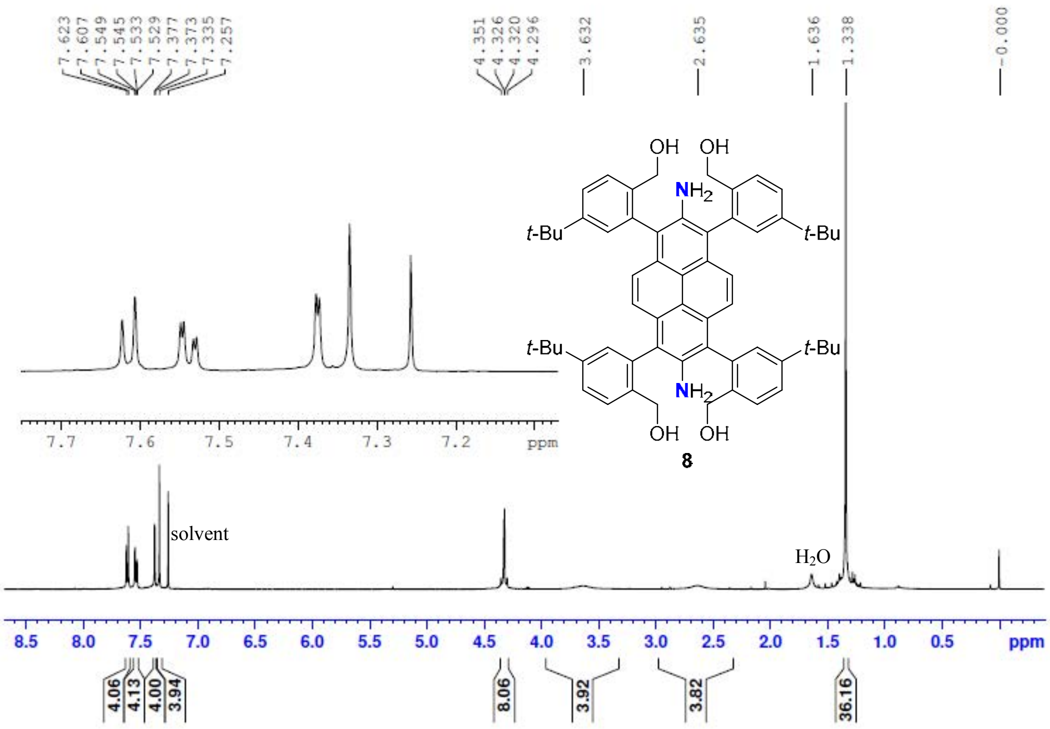

Supplementary Figure 5. ^{1}H NMR spectrum of **8** (500 MHz, $CDCl_3$, 297 K).

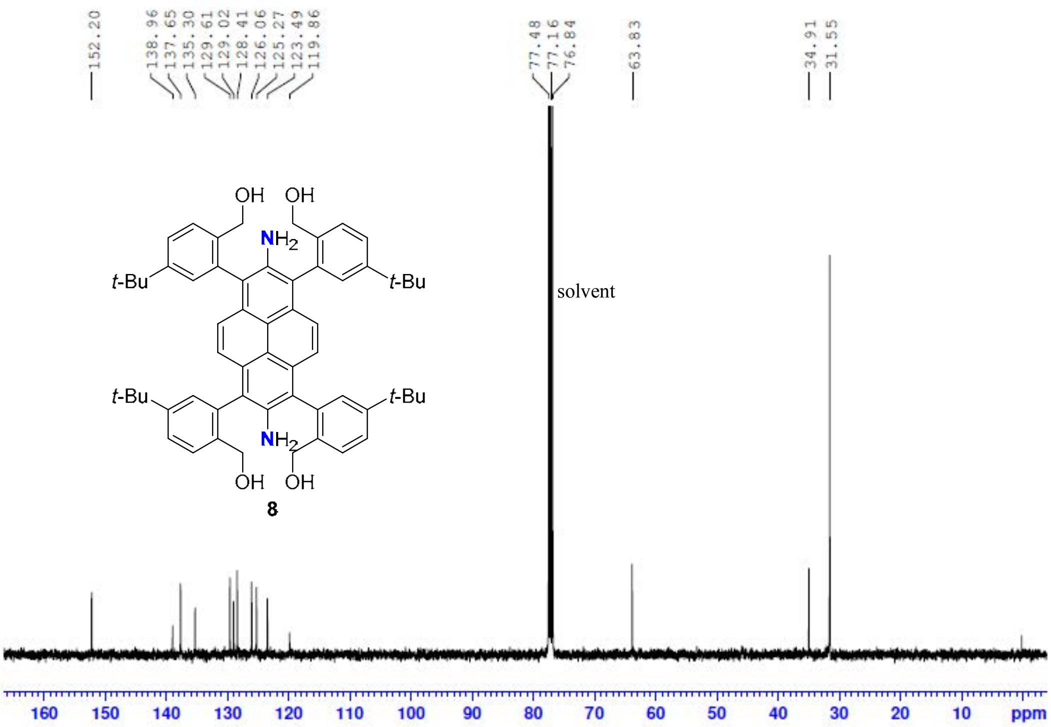

Supplementary Figure 6. ^{13}C NMR spectrum of **8** (126 MHz, $CDCl_3$, 297 K).

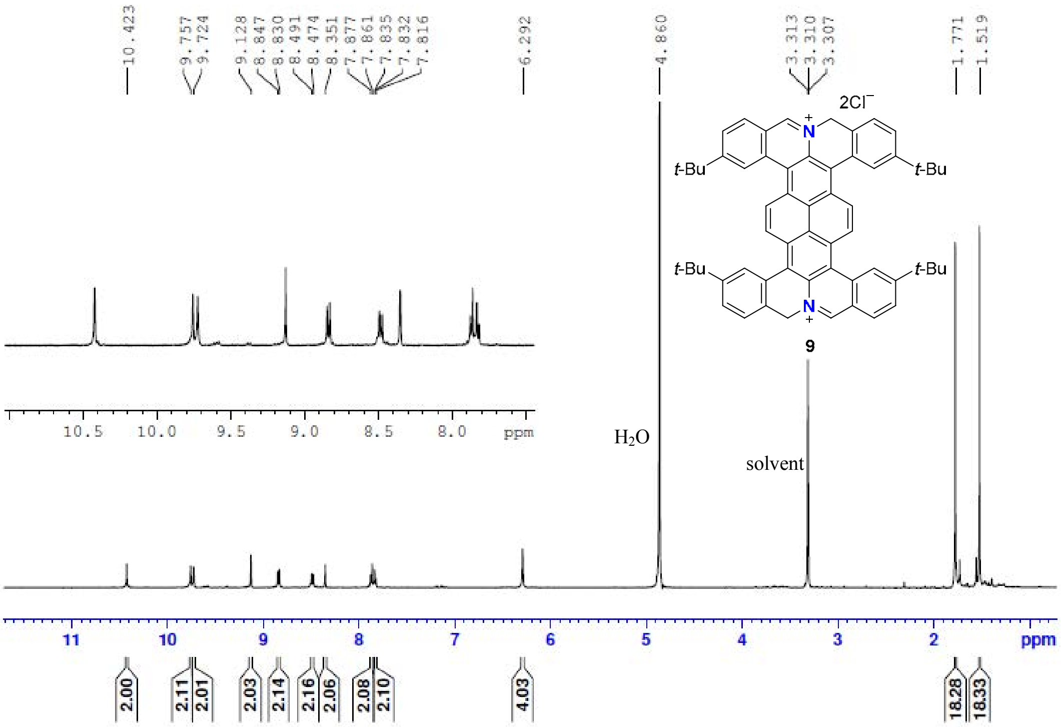

Supplementary Figure 7. ^{1}H NMR spectrum of **9** (500 MHz, CD_3OD, 297 K).

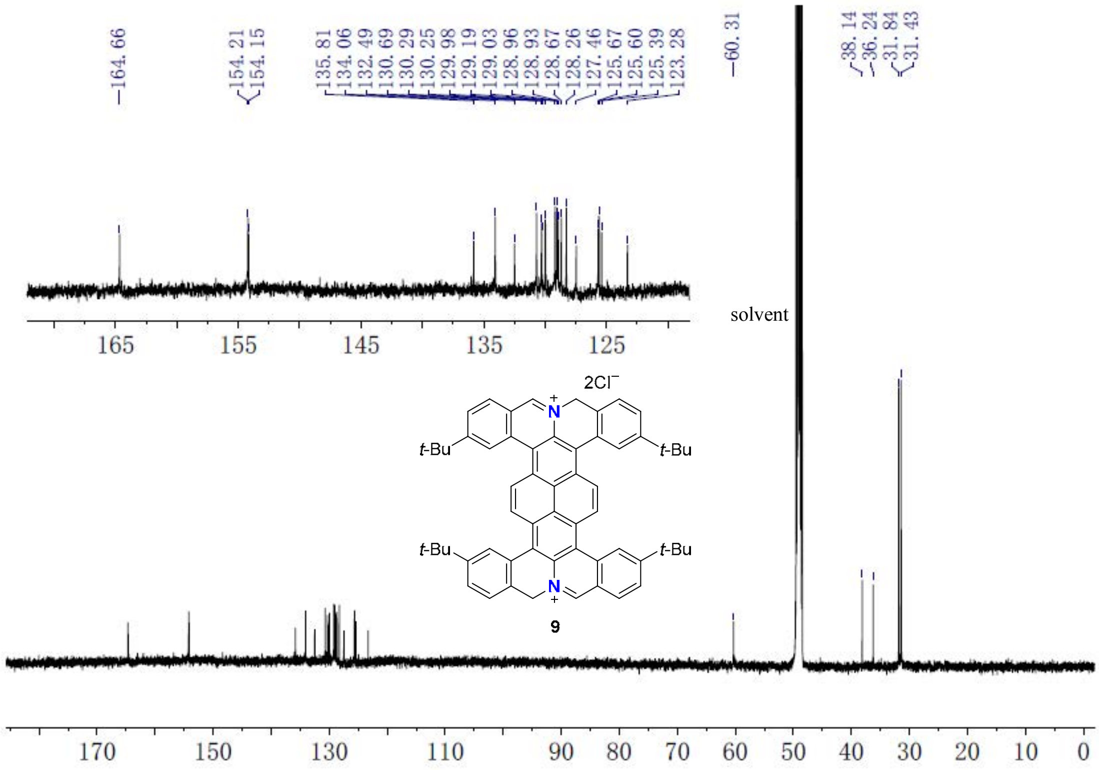

Supplementary Figure 8. ^{13}C NMR spectrum of **9** (126 MHz, CD_3OD, 297 K).

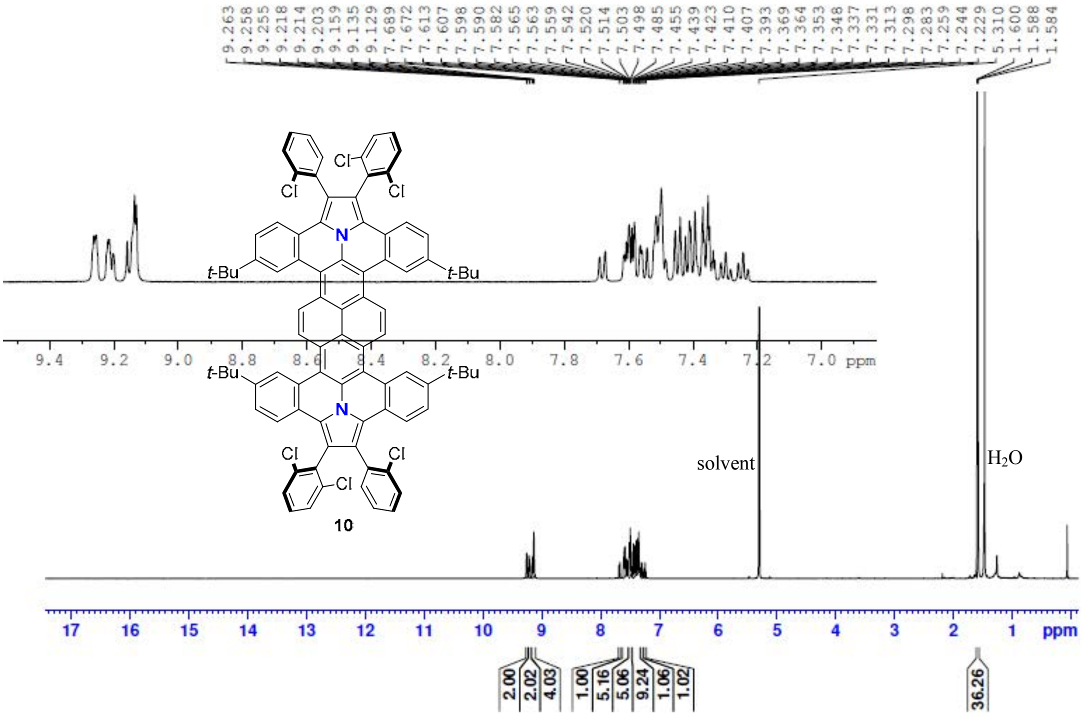

Supplementary Figure 9. ^{1}H NMR spectrum of **10** (500 MHz, CD_2Cl_2/CS_2, 297 K).

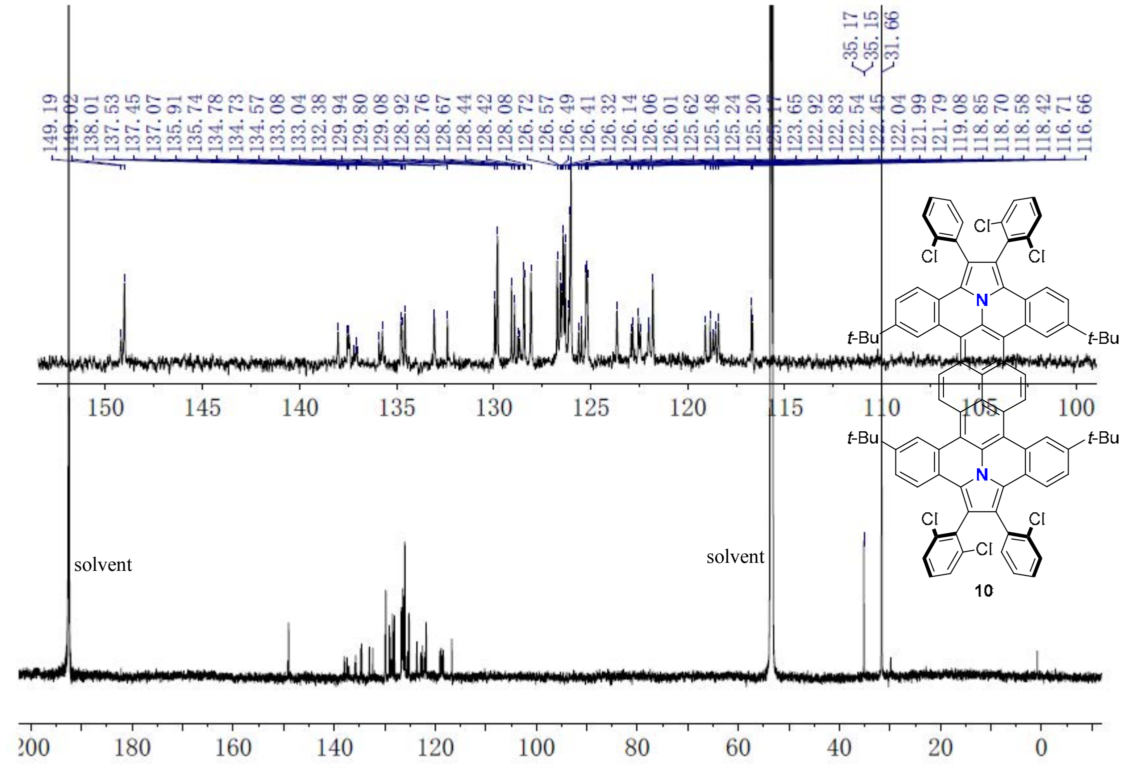

Supplementary Figure 10. ^{13}C NMR spectrum of **10** (201 MHz, CD_2Cl_2/CS_2, 298 K).

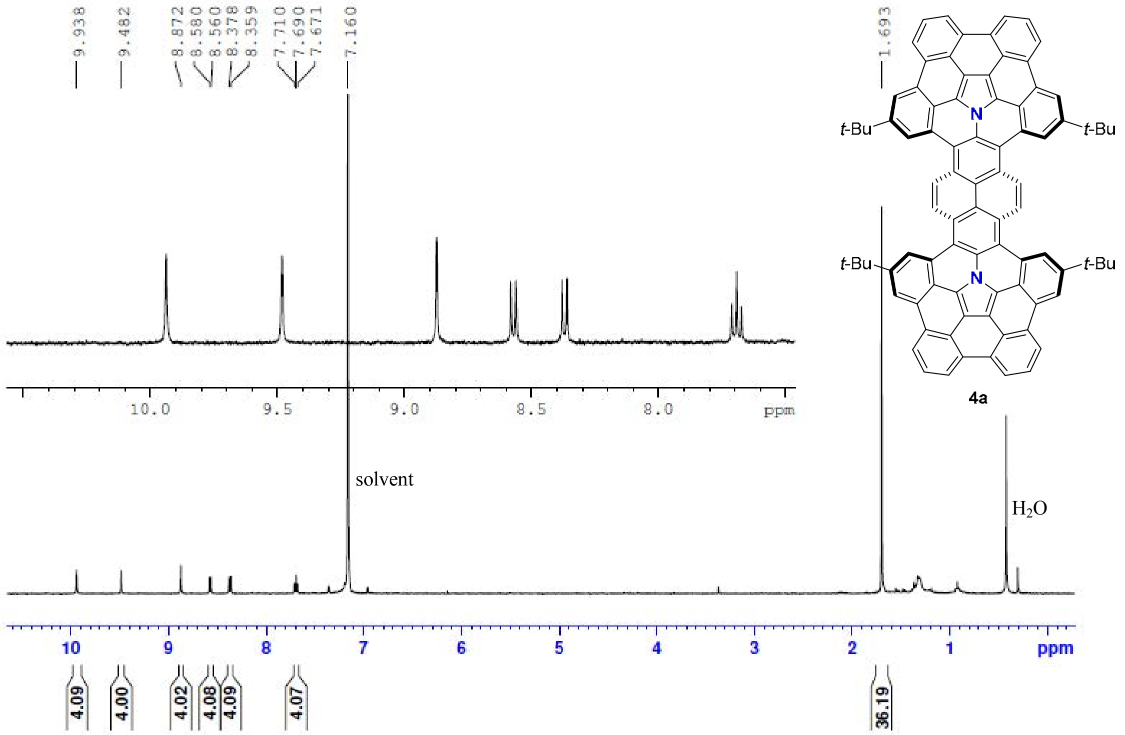

Supplementary Figure 11. ^{1}H NMR spectrum of **4a** (400 MHz, C_6D_6, 297 K).

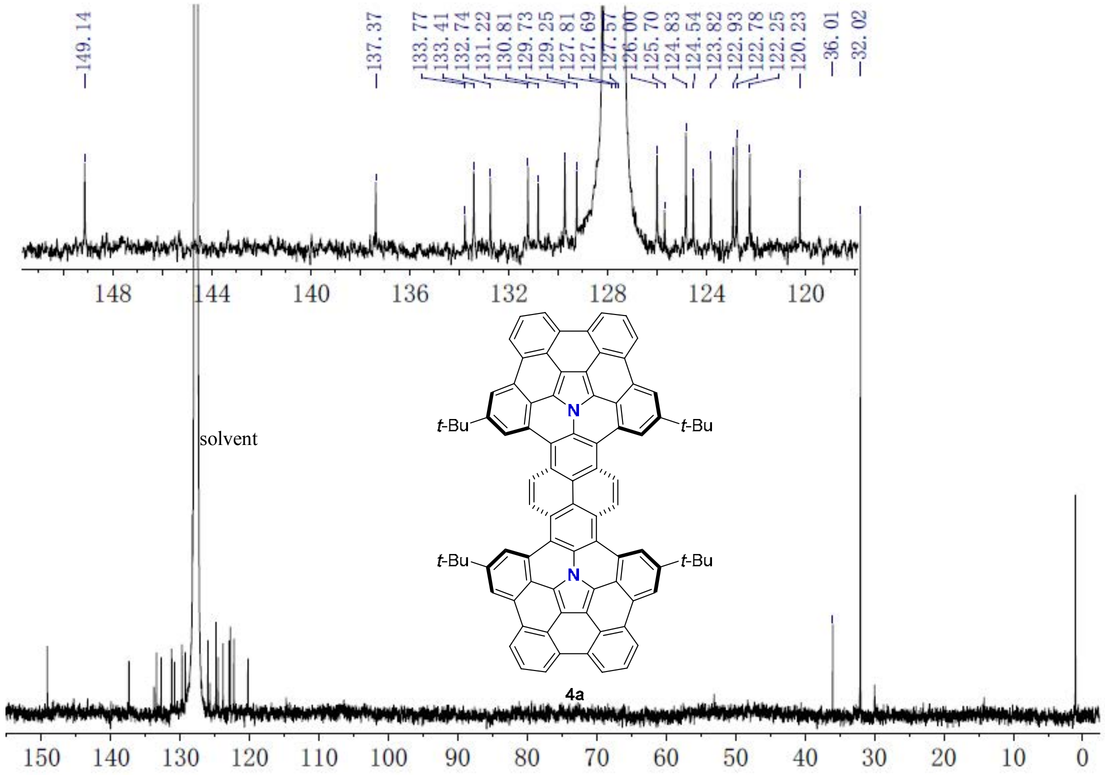

Supplementary Figure 12. ^{13}C NMR spectrum of **4a** (201 MHz, C_6D_6/CS_2, 298 K).

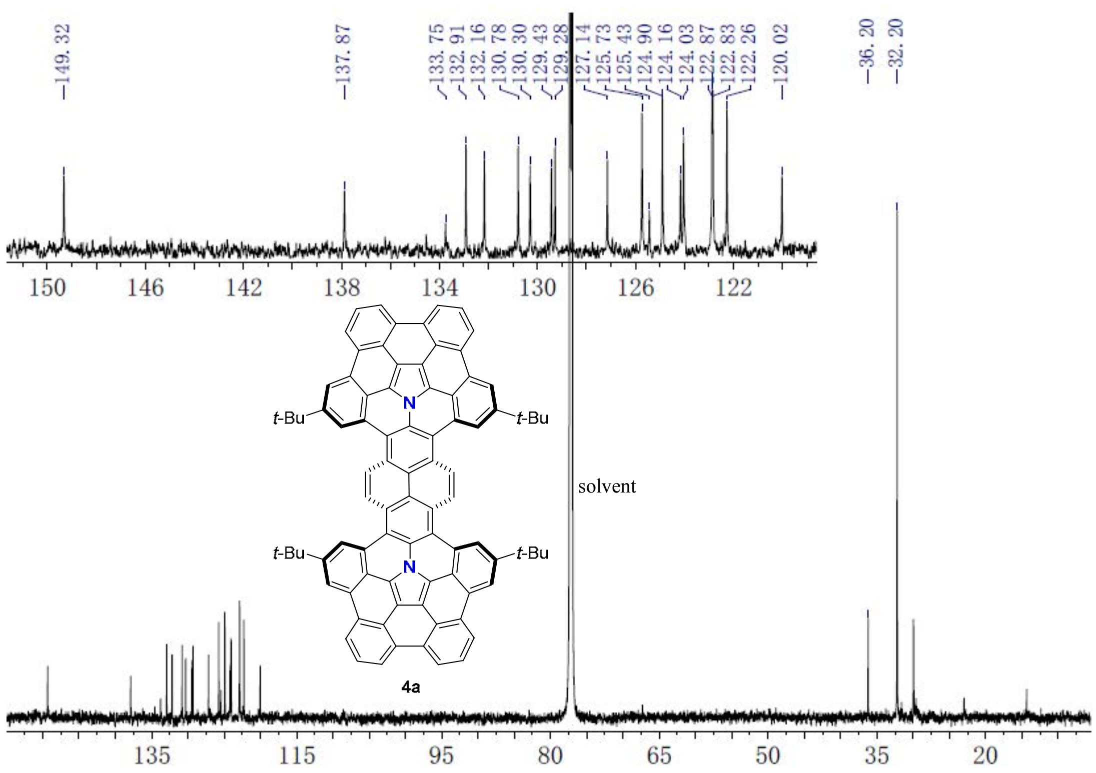

Supplementary Figure 13. ^{13}C NMR spectrum of **4a** (201 MHz, $CDCl_3/CS_2$, 298 K).

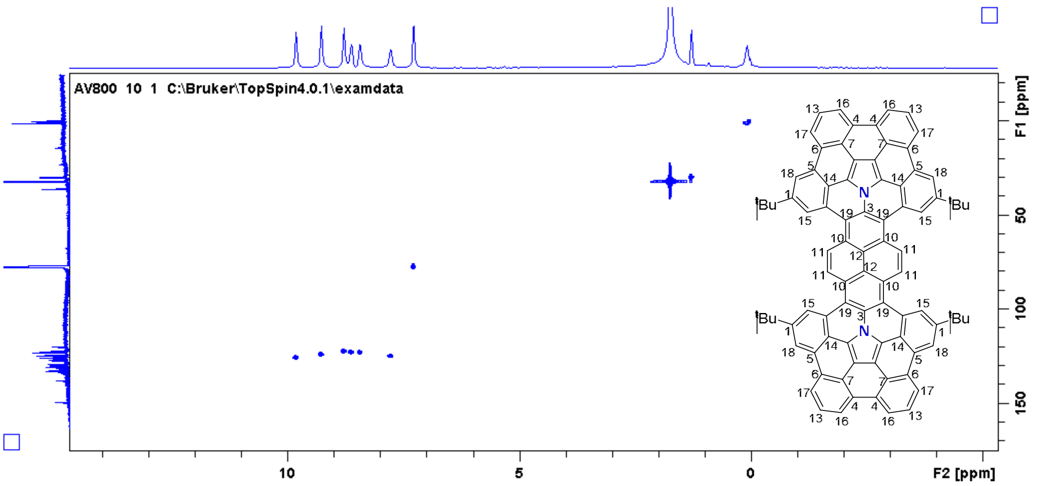

Supplementary Figure 14. HMQC spectrum of **4a** (201 MHz, $CDCl_3/CS_2$, 298 K).

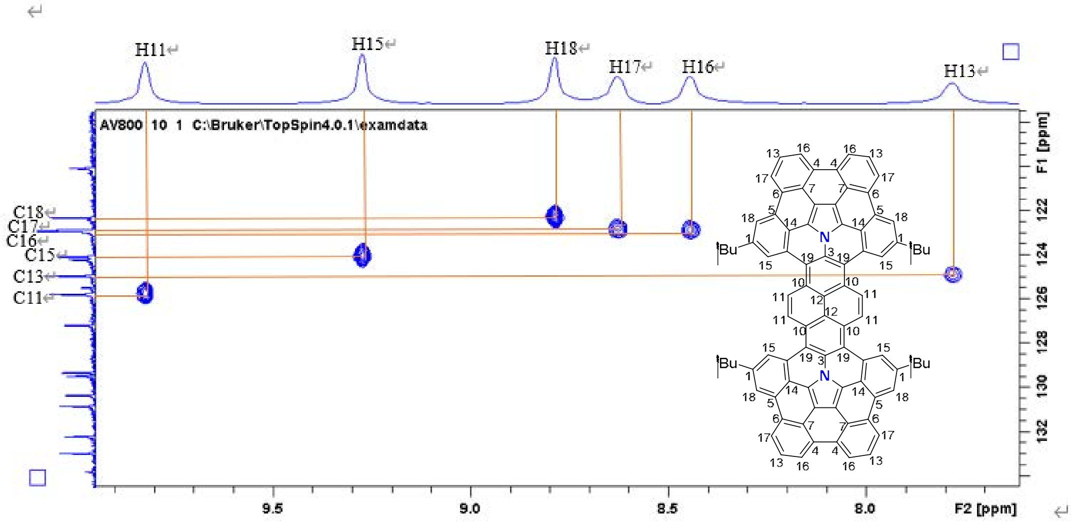

Supplementary Figure 15. Enlarge HMQC spectrum of **4a** (201 MHz, $CDCl_3/CS_2$, 298 K).

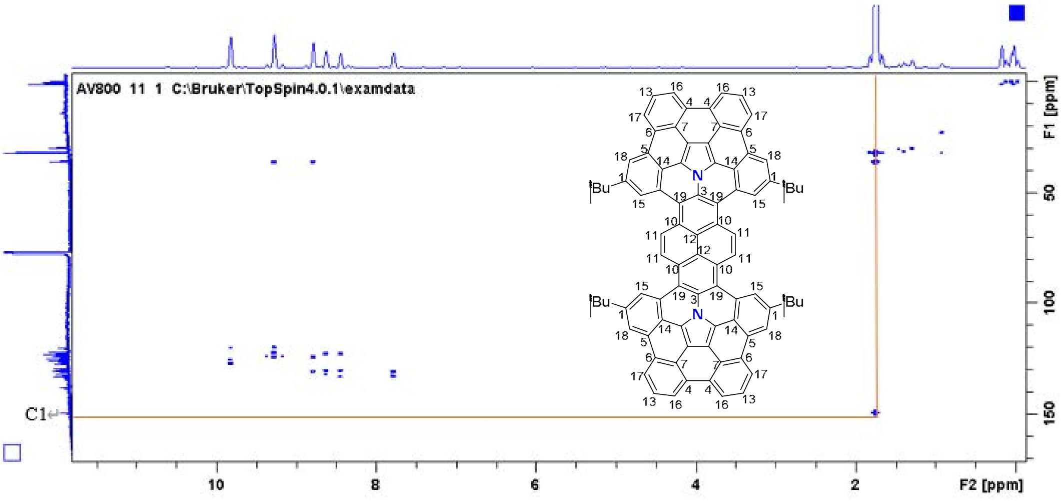

Supplementary Figure 16. HMBC spectrum of **4a** (201 MHz, $CDCl_3/CS_2$, 298 K).

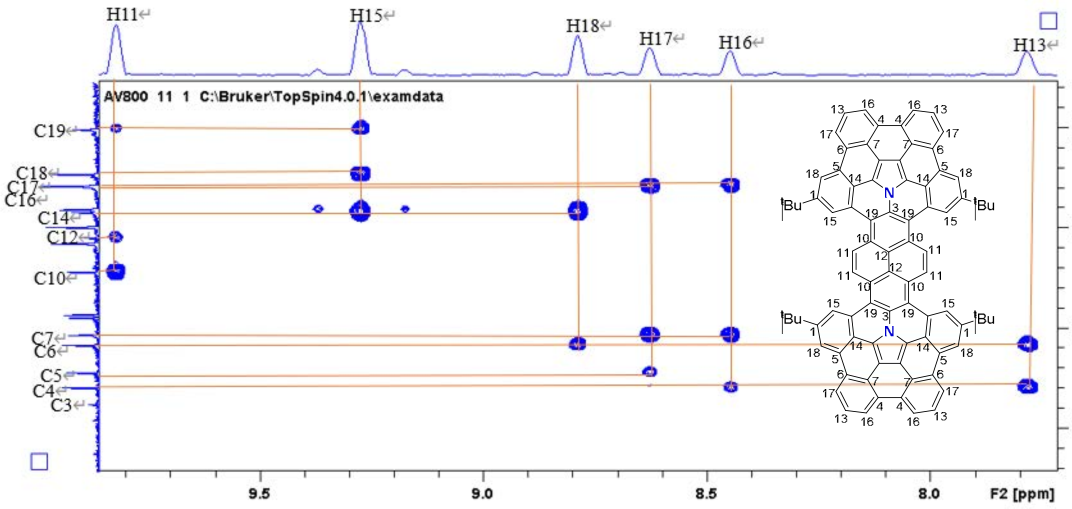

Supplementary Figure 17. Enlarge HMBC spectrum of **4a** (201 MHz, $CDCl_3/CS_2$, 298 K).

4. X-Ray Crystallographic Data

Single crystals suitable for X-ray analysis for **4a** was obtained by slow evaporation from a benzene/diethyl ether solution of **4a** under argon atmosphere at room temperature. A single crystal was mounted with mineral oil on a loop-type mount and transferred to the goniometer of a Bruker D8 Quest diffractometer. The radiation was performed with Multilayer Mirror-monochromated Incoatec microfocus source (λ =0.71073 Å). The structures were solved by direct method with SHELXT[4] and refined by full-matrix least-squares techniques against F_2 with SHELXL-2018/3.[4] The intensities were corrected for Lorentz and polarization effects. The non-hydrogen atoms were refined anisotropically. Hydrogen atoms were placed using AFIX instructions.

Supplementary Table 1. Crystal data and structure refinement for **4a**.

compound	**4a**
CCDC number	2103521
Molecular formula	$C_{100}H_{72}N_2$
Formula weight	1301.59
Temperature (K)	100(2)
Wavelength (Å)	0.71073
Crystal system	triclinic
Space group	*P*-1
Unit cell dimensions a (Å)	15.5009(10)
b (Å)	16.9360(9)
c (Å)	17.1394(9)
α (°)	63.3531(12)
β(°)	87.3698(13)
γ(°)	87.2850(14)
Volume (Å^3)	4015.5(4)
Z	2
Density (calculated) (mg·m^{-3})	1.076
Absorption coefficient (mm^{-1})	0.061
F(000)	1372
Crystal size (mm^3)	0.100×0.140×0.200
Theta range (°)	1.84 to 25.02
Reflections collected	14113
Min. and max. transmission	0.9880, 0.9940
Data / restraints / parameters	14113 / 2410 / 1152
Goodness-of-fit on F^2	1.021
Final *R* indices [$I > 2\sigma(I)$]	R_1 = 0.0858
	wR_2 = 0.1970
R indices (all data) [$I > 2\sigma(I)$]	R_1 = 0.1523
	wR_2 = 0.2339
Largest diff. peak and hole (e.Å^{-3})	0.225, -0.256

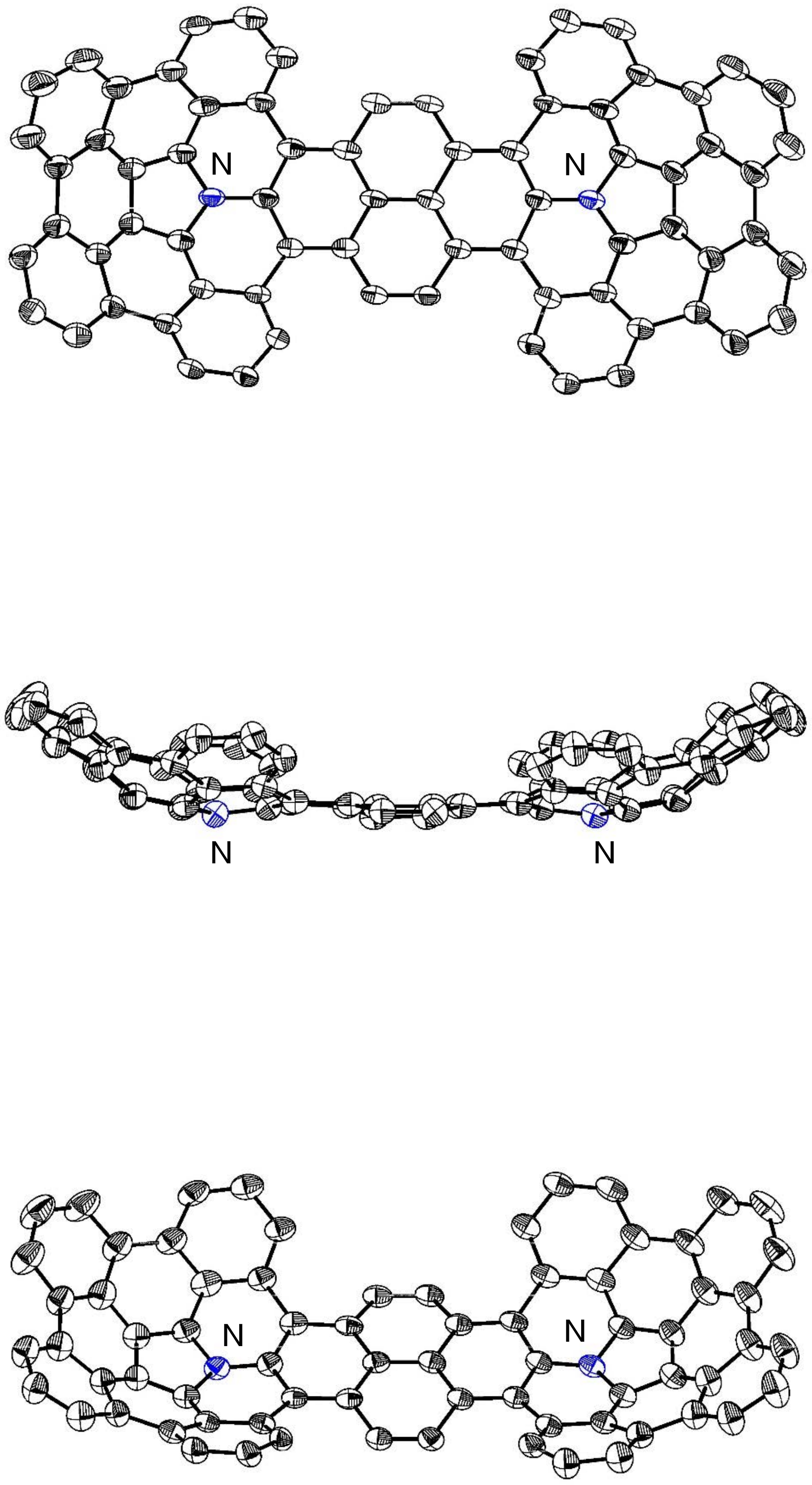

Supplementary Figure 18. X-ray structures of **4a** with thermal ellipsoids set at 50% probability. Solvent molecules, *tert*-butyl groups, and hydrogen atoms were omitted for clarity.

5. Optical Properties

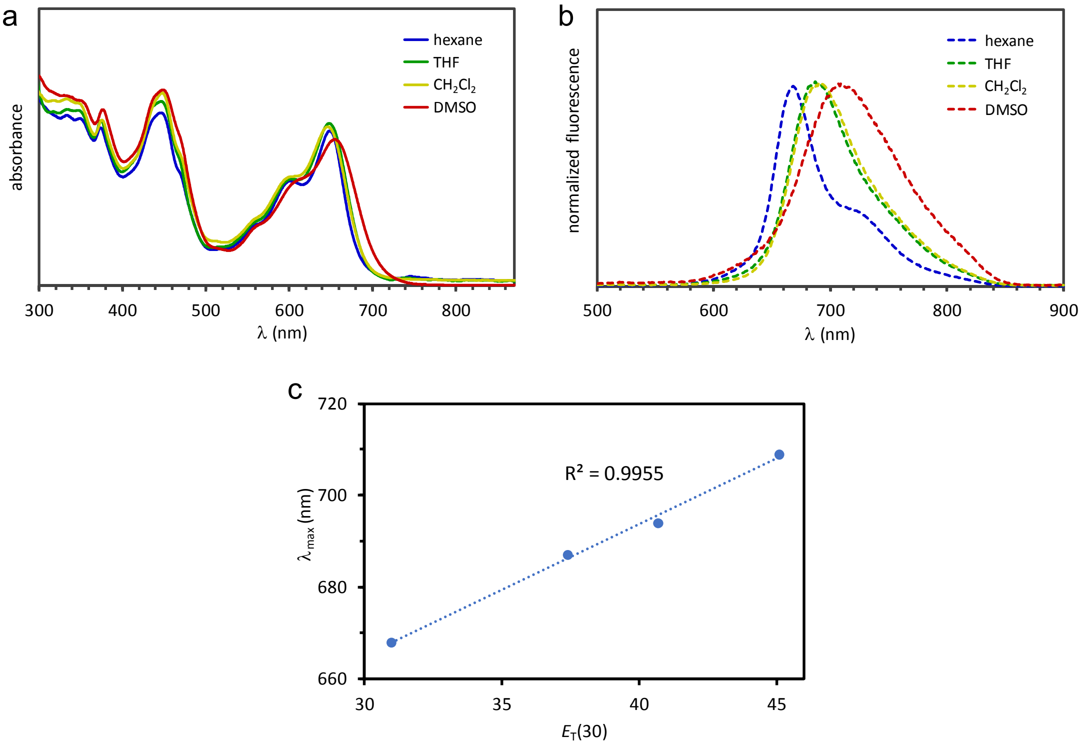

Supplementary Figure 19. a) Absorption spectra of **4a** in various solvents (1.0×10^{-5} M). b) Normalized emission spectrum of **4a** in various solvents (λ_{ex} = 450 nm, 1.0×10^{-6} M). c) Linear fitting of fluorescence λ_{max} against solvent polarity parameter $E_T(30)$.

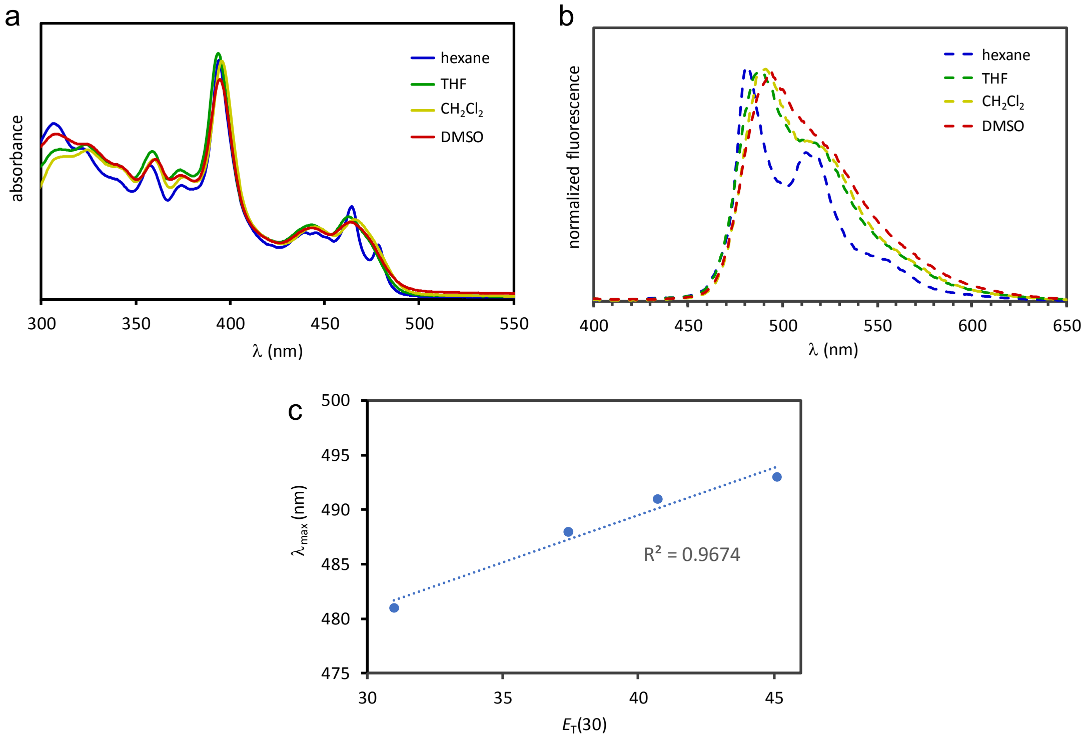

Supplementary Figure 20. a) Absorption spectra of APBC in various solvents (1.0×10^{-5} M). b) Normalized emission spectrum of APBC in various solvents (λ_{ex} = 320 nm, 1.0×10^{-6} M). c) Linear fitting of fluorescence λ_{max} against solvent polarity parameter $E_T(30)$.

Supplementary Table 2. Absorption wavelength, fluorescence wavelength, and quantum yield of **4a**.

Solvent	λ_{abs} (nm)[a]	λ_{fl} (nm)[b,c]	Φ (%)[c,d]
hexane	376, 447, 603, 650	668, 726	31
THF	376, 447, 604, 650	687	27
CH_2Cl_2	377, 447, 600, 650	694	22
DMSO	378, 451, 609, 658	709	15

[a] Concentration 1.0×10^{-5} M. [b] Concentration 1.0×10^{-6} M. [c] excited at 450 nm. [d] in reference to quinine sulfate in 0.1 M H_2SO_4 (λ_{ex}: 350 nm).

Note) Compound **4a** is not soluble in some polar solvents such as MeOH and acetonitrile.

6. Association with Fullerenes

To determine the association constant (K_a) of APBC and **4a** with C_{60} and C_{120}, fluorescence spectral titration analysis was carried out in 1,2-diclorobenzene. Stock solution **A** and **B** were prepared as shown in **Table S3**. Titration was performed by successive addition of solution **B** into 2.0 mL of solution **A**. Excitation wavelength of 320 nm and 450 nm was used for emission spectra of APBC and **4a**, respectively. K_a values were obtained by using the Bensi-Hildebrand equation as follows:

$$\frac{1}{\Delta Int_{max}} = \frac{1}{K_a \times \Delta\, Int_{max}} \times \frac{1}{[C_{60}/C_{120}]} + \frac{1}{\Delta Int_{max}}$$

Supplementary Table 3. Concentrations of stock solutions for each titration experiment in 1,2-diclorobenzene.

Host molecule	C_{60}			C_{120}		
	Solution **A**	Solution **B**		Solution **A**	Solution **B**	
	[host] (M)	[host] (M)	[C_{60}] (M)	[host] (M)	[host] (M)	[C_{120}] (M)
APBC	1.0×10^{-5}	1.0×10^{-5}	4.6×10^{-4}	1.5×10^{-5}	1.5×10^{-5}	4.6×10^{-4}
**4a**	1.5×10^{-5}	1.5×10^{-5}	9.3×10^{-3}	1.5×10^{-5}	1.5×10^{-5}	2.3×10^{-3}

Job plot of 4a and C_{120}

A stock solution of **4a** (1.0×10^{-5} M in 1,2-dichlorobenzene) and a stock solution of C_{120} (1.0×10^{-5} M in 1,2-dichlorobenzene) were prepared. The stock solutions were mixed with different ratios of **4a** and C_{120} (10:0, 9:1, 8:2, 7:3, 6:4, 5:5, 4:6, 3:7, 2:8, 1:9, 0:10), while the sum of the concentrations of **4a** and C_{120} was kept constant at 1.0×10^{-5} M.

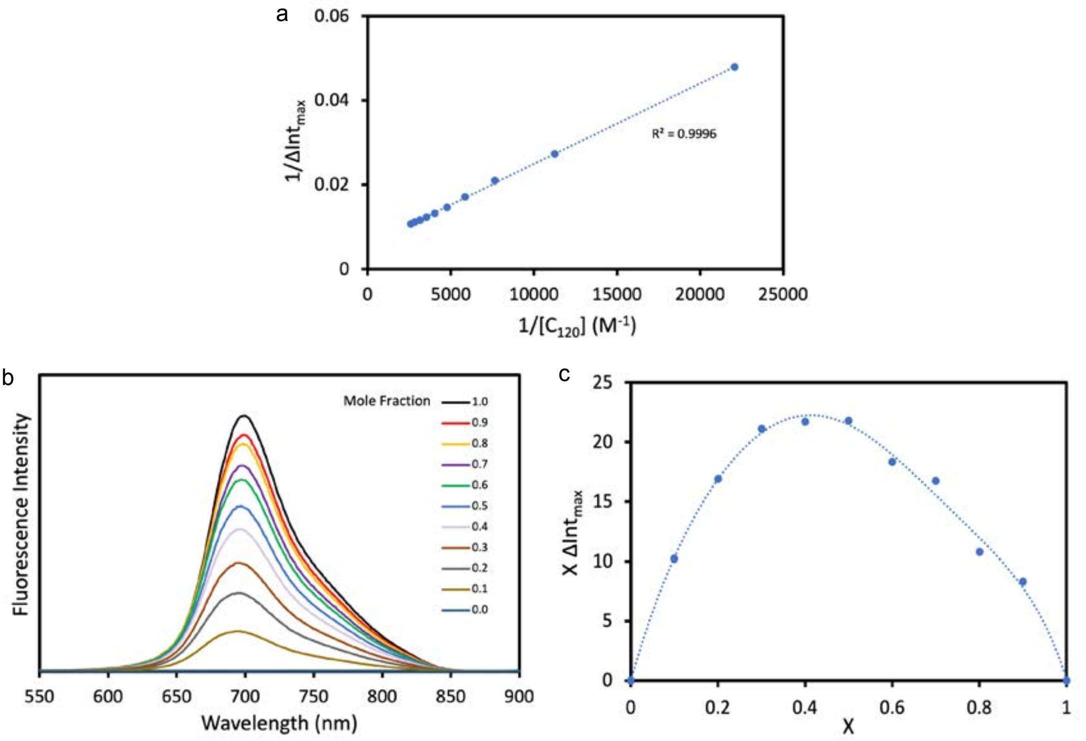

Supplementary Figure 21. a) Bensi-Hildebrand plot on titration of C_{120} into a solution of **4a** ($K_a = 2.9 \times 10^3$ M^{-1}). b) Fluorescence spectrum of **4a**–C_{120} at different mole fraction. c) Job's plot of **4a**–C_{120} (X = mole fraction).

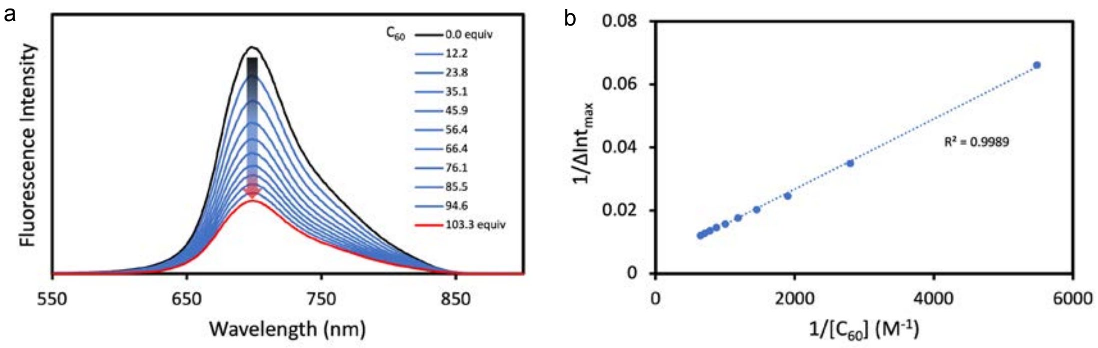

Supplementary Figure 22. a) Fluorescence spectrum of **4a** upon addition of 0–103 equiv of C_{60}. b) Bensi-Hildebrand plot on titration of C_{60} into a solution of **4a** (K_a= 4.5 × 10^2 M^{-1}).

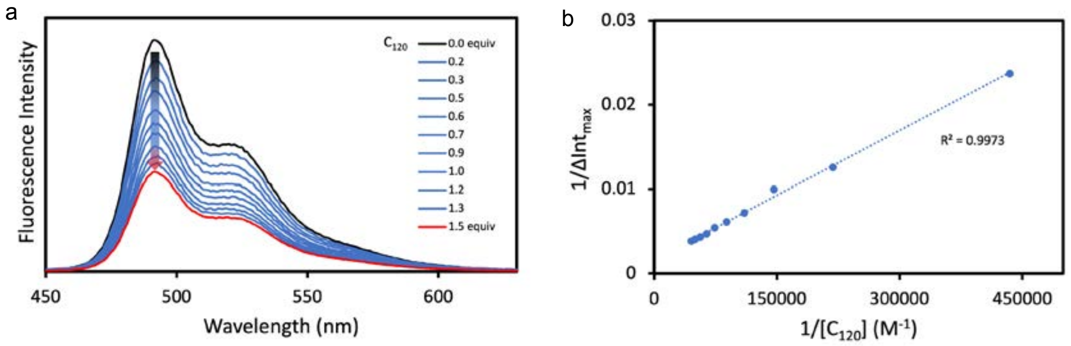

Supplementary Figure 23. a) Fluorescence spectrum of APBC upon addition of 0–1.5 equiv of C_{120}. b) Bensi-Hildebrand plot on titration of C_{120} into a solution of APBC (K_a = 3.2 × 10^4 M^{-1}).

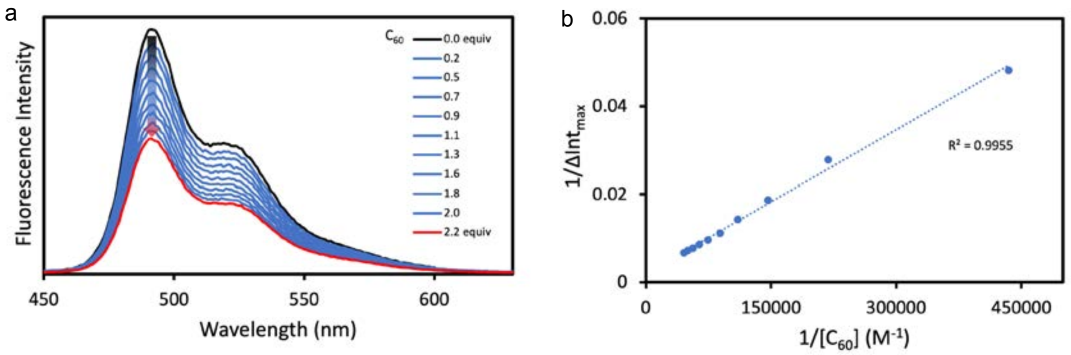

Supplementary Figure 24. a) Fluorescence spectrum of APBC upon addition of 0–2.2 equiv of C_{60}. b) Bensi-Hildebrand plot on titration of C_{60} into a solution of APBC (K_a = 2.0 × 10^4 M^{-1}).

7. Electrochemical Properties

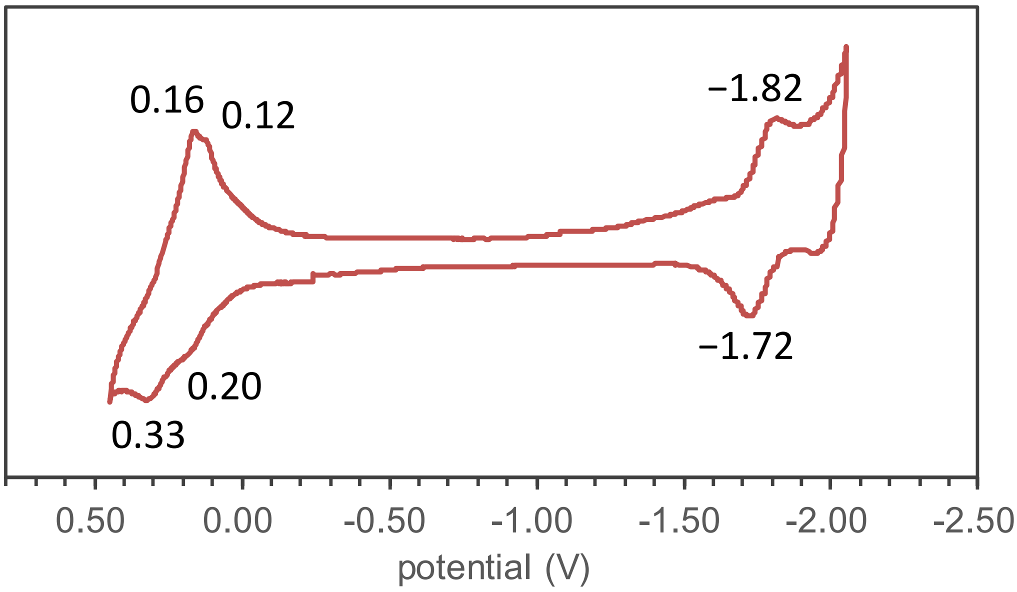

Supplementary Figure 25. Cyclic voltammogram of **4a** measured in THF with scan rate of 100 mV/s using with Bu_4PF_6 as an electrolyte, Ag/Ag^+ as a reference electrode, Pt as a working electrode, and Pt wire as a counter electrode. The potential was calibrated against Fc/Fc^+.

Note) The HOMO-LUMO gap of **4a** is determined by

$$\frac{0.12 + 0.20}{2} - \frac{-1.82 + (-1.72)}{2}$$
$$= 1.94\ \mathrm{V}$$

8. Theoretical Calculations

The computations were performed using workstation at High-Performance Center for Computational Science, Nanyang Technological University, Singapore. All the calculations were performed by using Gaussian 16 (revision A.03) program[5] by the B3LYP method[6] with the 6-31G(d) basis set[7] for structure optimization, vibrational frequency, time-dependent density functional theory (TD-DFT), NICS, and ACID calculations. Grimme's D3 dispersion correction was used to investigate the association of **4b** with C_{120} (**Section 8-7**). The geometries of **4b** were optimized without any symmetry assumptions. Each transition state structure was optimized without any symmetry assumptions and IRC calculations were also performed to check the transition states.

8-1. Conformation

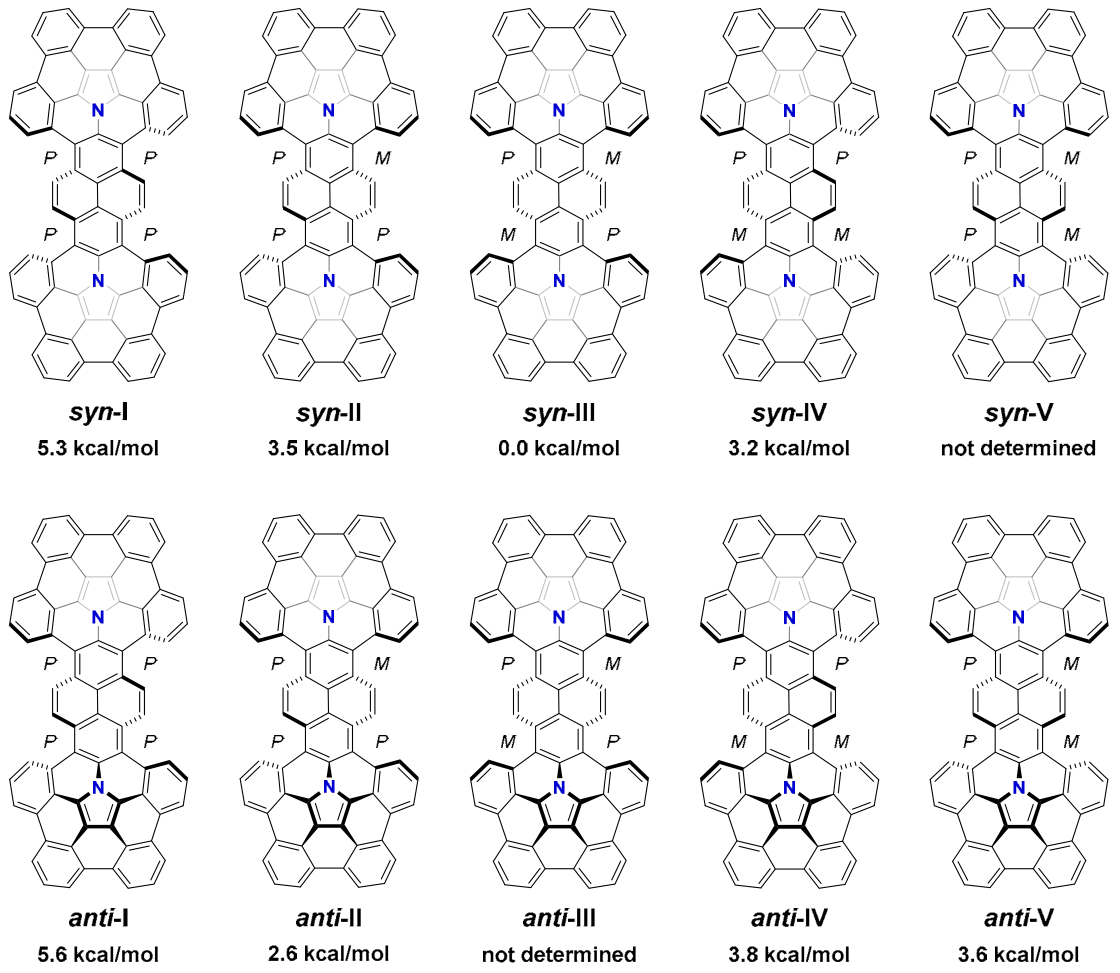

Supplementary Figure 26. Conformers and energies of **4b** relative to ***syn*-III**.

8-2. Helicene Flipping and Bowl Inversion

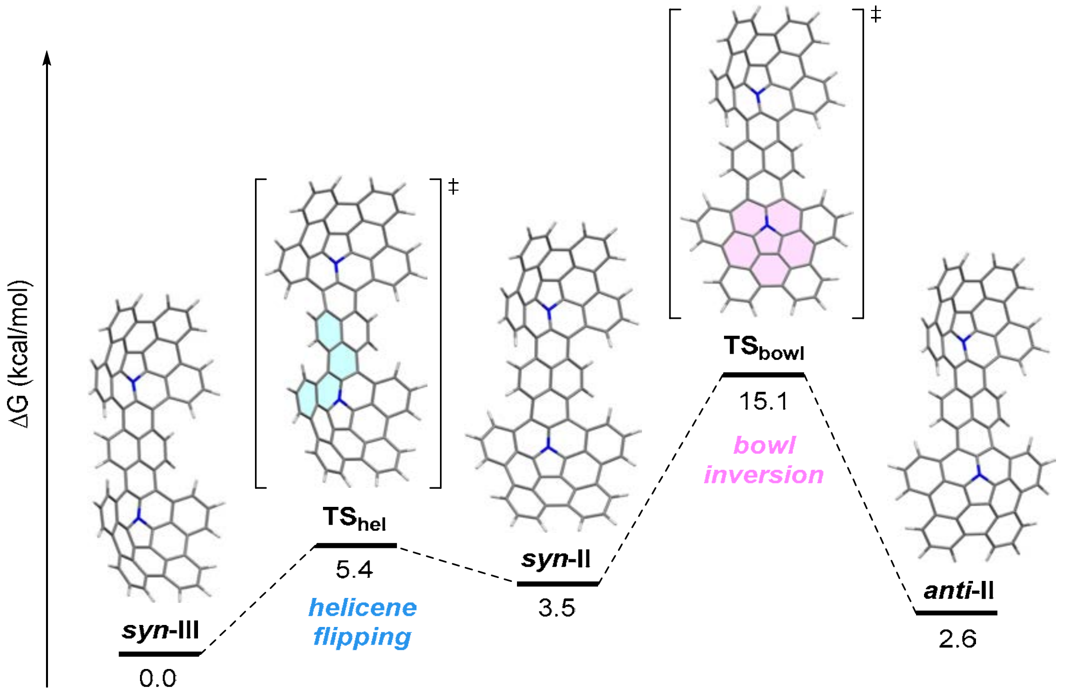

Supplementary Figure 27. Helicene flipping (**syn-III** to **syn-II**) via **TS$_{hel}$** and bowl inversion (***anti*-III** to ***anti*-II**) via **TS$_{bowl}$**.

8-3. Molecular Orbitals

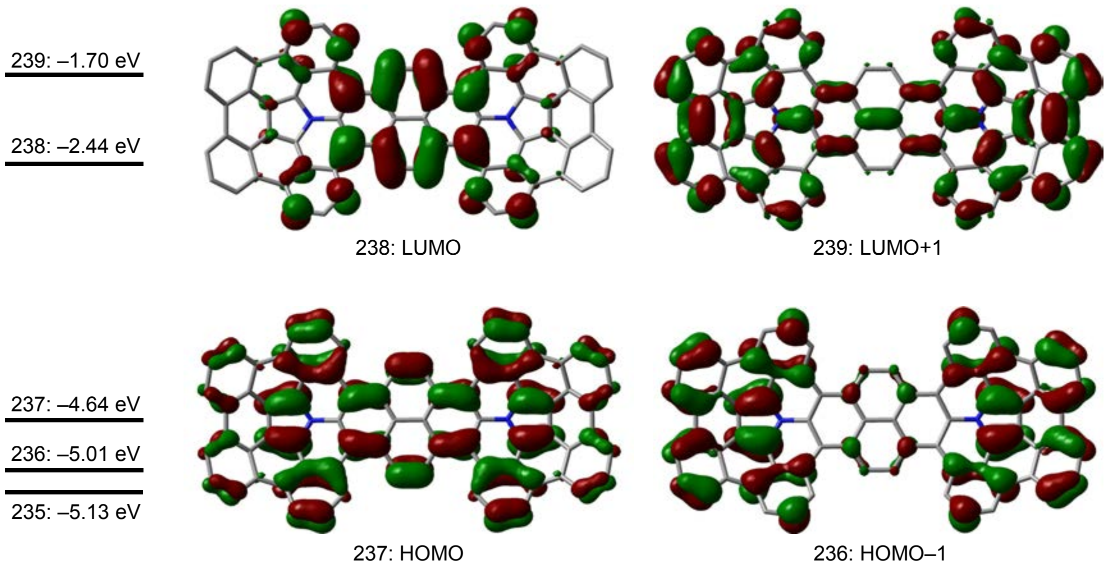

Supplementary Figure 28. Kohn-Sham molecular orbitals of **4**. Each MO is shown from the concave surface.

8-4. NICS Analysis

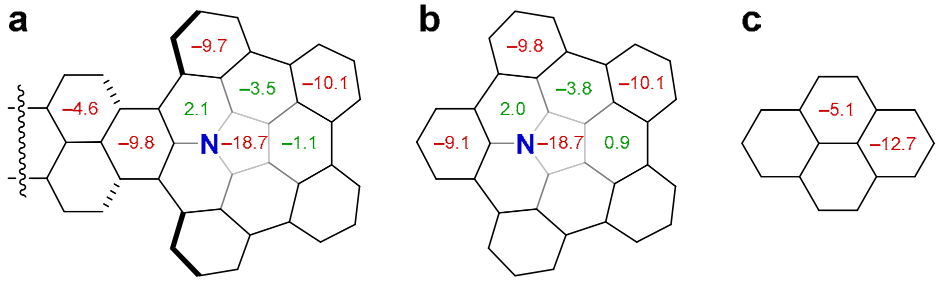

Supplementary Figure 29. NICS(0) values of (a) **4**, (b) APBC, and (c) pyrene.

8-5. ACID Plots

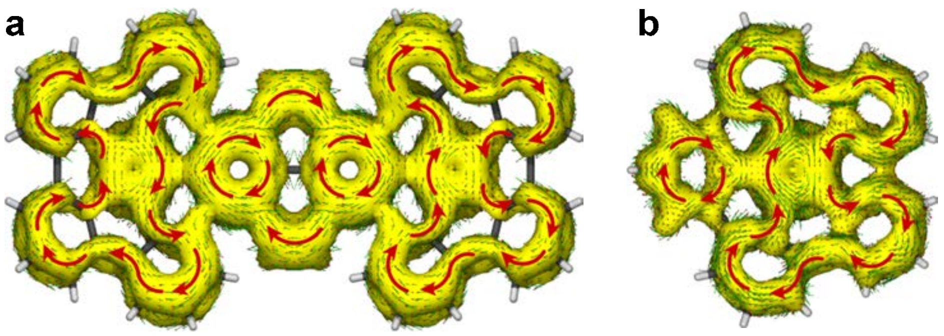

Supplementary Figure 30. ACID plot of (a) **4** and (b) APBC.

8-6. TD-DFT Calculations

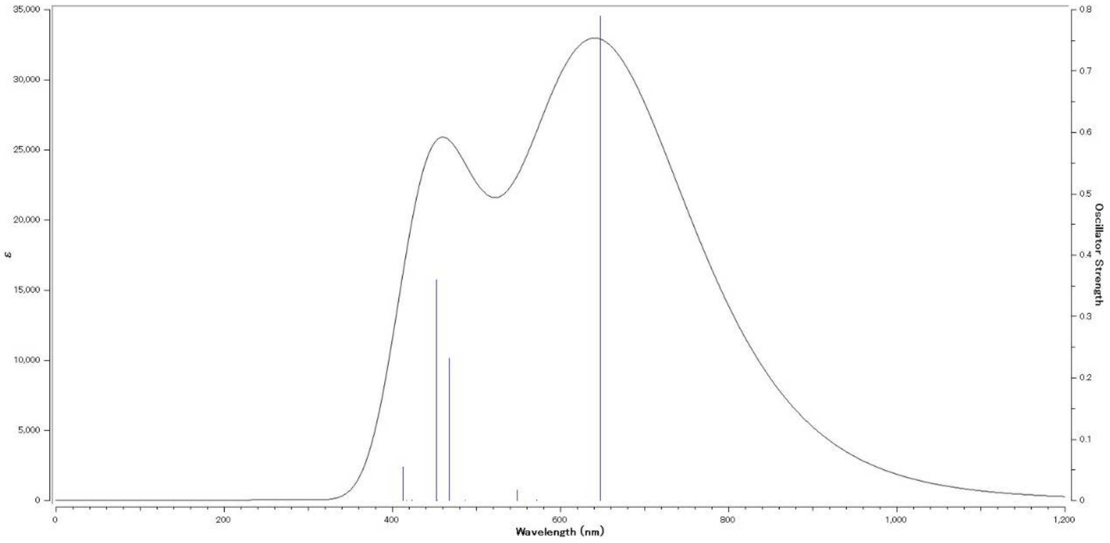

Supplementary Figure 31. Simulated UV-vis absorption spectrum of **4b.**

Supplementary Table 4. Selected wavelengths, oscillator strengths, major electronic transition of **4**.

Wavelength (λ)	Oscillator Strengths (*f*)	Transitions
679.09	0.7897	237→238 (0.69567)
571.25	0.0013	236→238 (0.69837)
548.46	0.0170	235→238 (0.64036)<br>237→239 (0.27239)
486.31	0.0000	234→238 (0.60681)<br>236→239 (0.13027)<br>237→240(0.32528)
467.42	0.2323	235→238 (−0.27042)<br>237→239 (0.63645)
452.96	0.0000	234→238 (−0.33724)<br>237→240 (0.60214)
452.30	0.3596	233→238 (0.69051)
423.13	0.0013	232→238 (0.11258)<br>234→239 (−0.19712)<br>235→240 (−0.27077)<br>237→241 (0.59917)
416.32	0.0000	236→239 (0.66629)<br>237→240 (−0.11697)
412.79	0.0544	236→240 (0.31881)<br>237→242 (0.53224)<br>237→244 (0.28700)

Note) 234: HOMO−3, 235: HOMO−2, 236: HOMO−1, 237: HOMO, 238: LUMO, 239: LUMO+1, 240: LUMO+2, 241: LUMO+3, 242: LUMO+4

8-7. Association of 4b and C_{120}

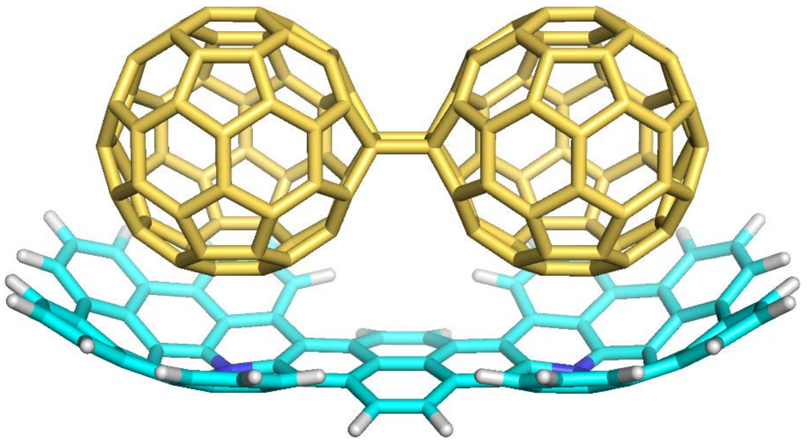

Supplementary Figure 32. One of the association modes of **4b** and C_{120}.

8-8 Cartesian Coordinates

***syn*-I**

C	-1.35046800	-1.31873600	-0.47727600
C	-2.76569000	-1.39102500	-0.34459300
C	-3.46110800	-0.24604400	-0.83321800
C	-2.91837400	1.06176600	-1.03909600
C	-1.51223000	1.15973500	-0.78474400
C	-0.72038300	-0.04068300	-0.66002000
C	-0.83049700	2.40206000	-0.62055500
C	0.52015900	2.47551500	-0.44306200
C	1.35046600	1.31873500	-0.47727600
C	0.72038200	0.04068300	-0.66001900
C	1.51222900	-1.15973500	-0.78474000
C	0.83049600	-2.40206100	-0.62055100
C	-0.52016100	-2.47551500	-0.44306000
C	-3.53152300	-2.48622500	0.33658800
C	-4.96202400	-2.45992300	0.26636700
C	-5.53708800	-1.47165900	-0.52164400
N	-4.81204200	-0.40590300	-1.01317600
C	2.76568900	1.39102500	-0.34459200
C	3.46110700	0.24604300	-0.83321700
C	2.91837300	-1.06176700	-1.03909300
C	-5.67139200	0.64673000	-1.17951400
C	-5.25952700	1.96404800	-1.24045800
C	-3.85129300	2.21002300	-1.33554800
C	3.53152200	2.48622500	0.33658900
C	4.96202300	2.45992200	0.26636900
C	5.53708600	1.47165900	-0.52164200
N	4.81204200	0.40590300	-1.01317600
C	5.67139200	-0.64672900	-1.17951600
C	5.25952700	-1.96404800	-1.24045700
C	3.85129300	-2.21002400	-1.33554400
C	-6.30261500	2.94657300	-1.11048600
C	-5.88519500	4.26558700	-1.34777300
C	-4.55070900	4.52742900	-1.67074800
C	-3.54498200	3.54595700	-1.65218100
C	-3.00633600	-3.43658000	1.22764700
C	-3.85933100	-4.28220800	1.95262500
C	-5.25101000	-4.16953500	1.91726700
C	-5.86556300	-3.19832300	1.10996900
C	6.30261600	-2.94657200	-1.11048400
C	5.88519600	-4.26558800	-1.34776600
C	4.55071000	-4.52743100	-1.67073800
C	3.54498200	-3.54596000	-1.65217200
C	3.00633400	3.43658000	1.22764900
C	3.85933000	4.28220800	1.95262700
C	5.25101000	4.16953500	1.91726900
C	5.86556200	3.19832300	1.10997100
C	6.94252700	-0.24179300	-0.84542500
C	6.85902600	1.08377800	-0.41759100
C	-6.85902800	-1.08377800	-0.41759400
C	-6.94252800	0.24179400	-0.84542600
C	-7.74295600	-1.62059700	0.52102800
C	-8.89714900	-0.82940900	0.84971800
C	-8.99873500	0.59113200	0.34929200
C	-7.92632700	1.13692200	-0.42985000
C	-9.97048300	1.52204100	0.73609200
C	-9.82067200	2.88267400	0.41536900
C	-8.68495600	3.39416600	-0.22094300
C	-7.65878400	2.52738700	-0.63851400
C	-7.29324400	-2.77177500	1.24097300
C	-8.21330500	-3.28782100	2.17220700
C	-9.42711000	-2.63319400	2.39834300
C	-9.75420400	-1.40786800	1.79317200
C	7.74295600	1.62059700	0.52102900
C	8.89714900	0.82941000	0.84971800
C	8.99873700	-0.59113100	0.34929000
C	7.92632700	-1.13692100	-0.42985100
C	9.97048900	-1.52203800	0.73608200
C	9.82067800	-2.88267100	0.41535800
C	8.68496000	-3.39416300	-0.22095100
C	7.65878500	-2.52738600	-0.63851600
C	7.29324300	2.77177500	1.24097500
C	8.21330400	3.28782100	2.17221000
C	9.42710800	2.63319200	2.39834700
C	9.75420200	1.40786600	1.79317500
H	-1.39780600	3.31835000	-0.58140300
H	0.97965700	3.45019900	-0.34243500
H	1.39780500	-3.31835000	-0.58139400
H	-0.97965900	-3.45019800	-0.34243300
H	-6.59012800	5.09053000	-1.29859100
H	-4.26319200	5.54697400	-1.91277900
H	-2.54181500	3.85224200	-1.91467100
H	-1.94327500	-3.49502200	1.42308500
H	-3.41255900	-5.01217500	2.62224800
H	-5.84517800	-4.80131200	2.57093500
H	6.59012900	-5.09053000	-1.29858200

H	4.26319200	-5.54697700	-1.91276400
H	2.54181500	-3.85224700	-1.91465800
H	1.94327400	3.49502000	1.42308800
H	3.41255900	5.01217600	2.62224900
H	5.84517700	4.80131200	2.57093700
H	-10.83437600	1.21723900	1.32029900
H	-10.59495000	3.57645600	0.73195900
H	-8.59355100	4.46941800	-0.34791000
H	-7.98118900	-4.17582300	2.75333100
H	-10.11835900	-3.05402700	3.12369200
H	-10.66306400	-0.90009900	2.10383900
H	10.83438400	-1.21723600	1.32028500
H	10.59495900	-3.57645100	0.73194300
H	8.59355800	-4.46941500	-0.34792100
H	7.98118800	4.17582100	2.75333500
H	10.11835600	3.05402400	3.12369700
H	10.66306100	0.90009600	2.10384400

***syn*-II**

C	-1.36727600	-1.27741600	-0.35865500
C	-2.79251400	-1.32209100	-0.29247700
C	-3.44133400	-0.11070400	-0.66532300
C	-2.85228700	1.18646400	-0.70582000
C	-1.42837400	1.19740800	-0.74361300
C	-0.68397500	-0.03191200	-0.60189300
C	-0.69866400	2.38841400	-1.04263700
C	0.66107900	2.42613700	-1.00545800
C	1.43517500	1.25683700	-0.74756000
C	0.76030000	-0.01005800	-0.68307900
C	1.51952400	-1.23899200	-0.66163700
C	0.78810200	-2.43291900	-0.39714600
C	-0.56759800	-2.45210800	-0.25850100
C	-3.63942100	-2.46470500	0.20205700
C	-5.05772100	-2.40109800	-0.00450900
C	-5.55451900	-1.29344800	-0.68286500
N	-4.78042300	-0.18650700	-0.93839300
C	2.85025000	1.31167800	-0.64750600
C	3.51566800	0.07968600	-0.91265600
C	2.93461600	-1.22712500	-0.90159400
C	-5.60517400	0.90771200	-1.04888400
C	-5.16448200	2.19714200	-0.77127100
C	-3.75262700	2.38791400	-0.60699400
C	3.63612200	2.50400900	-0.19252700
C	5.06491200	2.42755100	-0.22790600
C	5.61963000	1.29326200	-0.80372700
N	4.87164600	0.17345600	-1.10201900
C	5.70467700	-0.91131700	-1.05927700
C	5.25746400	-2.20715700	-0.89135700
C	3.84412100	-2.43461100	-0.97781700
C	-6.19228100	3.14894300	-0.44086400
C	-5.72457000	4.43040600	-0.10639000
C	-4.35560400	4.67151900	0.01099300
C	-3.38637600	3.67532000	-0.18055400
C	-3.22178400	-3.52343300	1.02565400
C	-4.14719800	-4.44426700	1.53934800
C	-5.52431200	-4.31774600	1.35734700
C	-6.04512800	-3.23854700	0.62533600
C	6.27501300	-3.17251600	-0.56746800
C	5.83289600	-4.50414400	-0.58056400
C	4.50077100	-4.79026900	-0.88934600
C	3.51882100	-3.80088200	-1.06887300
C	3.12366800	3.63021100	0.47200000
C	3.99150100	4.57803600	1.03549200
C	5.37942900	4.41848200	1.06056000
C	5.97780700	3.29143100	0.47318800
C	6.98242400	-0.48267900	-0.78504500
C	6.92930600	0.90040900	-0.60946700
C	-6.87638200	-0.89694500	-0.65589900
C	-6.90809900	0.48295700	-0.88543300
C	-7.85098700	-1.56237600	0.09153300
C	-9.03483200	-0.82106400	0.42131400
C	-9.06889500	0.66461700	0.17390000
C	-7.91699200	1.31106600	-0.38724100
C	-10.06198400	1.53228400	0.64332000
C	-9.86295900	2.92268200	0.61895800
C	-8.65505700	3.50447300	0.22527800
C	-7.60621100	2.70096900	-0.25878700
C	-7.47941900	-2.82001300	0.66149800
C	-8.49526400	-3.46965400	1.38674300
C	-9.73103200	-2.84645400	1.57897300
C	-9.99199900	-1.53340700	1.15322200
C	7.81619200	1.58201700	0.22672700
C	8.94421400	0.83763400	0.71629000
C	9.01239000	-0.65377500	0.49062500
C	7.93678800	-1.30836200	-0.19493700
C	9.95083400	-1.52103000	1.06297900
C	9.76698400	-2.91342900	0.99726100
C	8.62768800	-3.50576500	0.44282200

C	7.63353900	-2.70595600	-0.14945800
C	7.39034300	2.85945500	0.70966300
C	8.31323600	3.51747400	1.54354700
C	9.50556500	2.88611800	1.90854800
C	9.80595400	1.56126700	1.54882500
H	-1.23722600	3.26670600	-1.37069000
H	1.17721500	3.34123500	-1.27242300
H	1.31410000	-3.36392600	-0.27213700
H	-1.04839700	-3.41033100	-0.13108400
H	-6.42095700	5.22823400	0.13382600
H	-4.02422500	5.65682400	0.32757300
H	-2.36245600	3.91293200	0.07425100
H	-2.19260000	-3.61850900	1.34338600
H	-3.77463900	-5.25460500	2.16015000
H	-6.18658900	-5.02343400	1.84980300
H	6.51716900	-5.32129500	-0.37084100
H	4.19313900	-5.83000700	-0.96019600
H	2.52023500	-4.13602900	-1.30929000
H	2.05774000	3.75949900	0.61397200
H	3.55798300	5.44155100	1.53277100
H	5.98136200	5.14996700	1.59180600
H	-10.98204400	1.14523600	1.07217100
H	-10.65677000	3.56741200	0.98665700
H	-8.53152700	4.57830600	0.33236800
H	-8.32517800	-4.44446200	1.83480100
H	-10.49825600	-3.37230400	2.14099700
H	-10.93176700	-1.06997400	1.44004700
H	10.81414000	-1.13531400	1.59825100
H	10.51590500	-3.55535100	1.45347400
H	8.50723300	-4.58314300	0.51587400
H	8.09863100	4.50399800	1.94459400
H	10.19971300	3.41793200	2.55383500
H	10.69747000	1.09856300	1.96303800

***syn*-III**

C	1.43888200	-1.25134300	-0.70115400
C	2.86247000	-1.27179100	-0.64506800
C	3.48649100	0.00000100	-0.79095700
C	2.86247100	1.27179400	-0.64506700
C	1.43888400	1.25134600	-0.70115400
C	0.72299000	0.00000200	-0.70454000
C	0.68061500	2.44475700	-0.89850900
C	-0.68061400	2.44475700	-0.89851100
C	-1.43888400	1.25134700	-0.70115900

C	-0.72299000	0.00000300	-0.70454400
C	-1.43888300	-1.25134200	-0.70115900
C	-0.68061400	-2.44475200	-0.89851300
C	0.68061400	-2.44475200	-0.89851200
C	3.72091800	-2.46891000	-0.34446100
C	5.14320900	-2.33490900	-0.46769200
C	5.63126400	-1.11540900	-0.92181200
N	4.83493400	0.00000100	-1.02740700
C	-2.86247100	1.27179500	-0.64507500
C	-3.48649200	0.00000100	-0.79096400
C	-2.86247000	-1.27179100	-0.64507500
C	5.63126400	1.11540800	-0.92180600
C	5.14321000	2.33491000	-0.46768800
C	3.72091900	2.46891300	-0.34446000
C	-3.72092000	2.46891200	-0.34446400
C	-5.14321100	2.33490800	-0.46768900
C	-5.63126500	1.11540800	-0.92180700
N	-4.83493500	0.00000000	-1.02741000
C	-5.63126400	-1.11540800	-0.92181100
C	-5.14320800	-2.33490700	-0.46769000
C	-3.72091800	-2.46890800	-0.34446300
C	6.12899000	3.24685500	0.04964000
C	5.61091800	4.45560000	0.54287000
C	4.23129500	4.65200200	0.61910800
C	3.29851000	3.67572000	0.23719000
C	3.29850700	-3.67571500	0.23719100
C	4.23129000	-4.65199900	0.61910700
C	5.61091400	-4.45560000	0.54286600
C	6.12898700	-3.24685700	0.04963600
C	-6.12898500	-3.24685400	0.04964300
C	-5.61091100	-4.45559500	0.54287600
C	-4.23128700	-4.65199300	0.61911600
C	-3.29850400	-3.67571000	0.23719400
C	-3.29850900	3.67571600	0.23719000
C	-4.23129400	4.65199800	0.61911000
C	-5.61091700	4.45559700	0.54287200
C	-6.12899000	3.24685300	0.04964300
C	-6.93826000	-0.69925600	-0.77103700
C	-6.93826000	0.69925400	-0.77103400
C	6.93826000	-0.69925600	-0.77103700
C	6.93825900	0.69925500	-0.77103300
C	7.90084900	-1.45815000	-0.10181000
C	9.04552300	-0.75357900	0.40174500
C	9.04552400	0.75357500	0.40174600

C	7.90084900	1.45814700	-0.10180700
C	9.99161100	1.55552500	1.05079800
C	9.75383000	2.92722200	1.23940900
C	8.54894000	3.54012500	0.88508300
C	7.54534500	2.80343500	0.22945300
C	7.54534300	-2.80343800	0.22944900
C	8.54893600	-3.54012800	0.88508000
C	9.75382700	-2.92722600	1.23940800
C	9.99160900	-1.55553000	1.05079900
C	-7.90085100	1.45814600	-0.10180800
C	-9.04552600	0.75357400	0.40174200
C	-9.04552600	-0.75358000	0.40174100
C	-7.90084900	-1.45815000	-0.10181000
C	-9.99161100	-1.55553100	1.05079500
C	-9.75382800	-2.92722600	1.23940700
C	-8.54893600	-3.54012800	0.88508200
C	-7.54534200	-2.80343600	0.22945200
C	-7.54534500	2.80343300	0.22945400
C	-8.54893900	3.54012200	0.88508500
C	-9.75383100	2.92722000	1.23940900
C	-9.99161200	1.55552400	1.05079600
H	1.20306600	3.36044500	-1.14285500
H	-1.20306300	3.36044600	-1.14285800
H	-1.20306400	-3.36044100	-1.14286100
H	1.20306400	-3.36044100	-1.14286100
H	6.27310600	5.22369100	0.93125000
H	3.85930500	5.57613700	1.05326000
H	2.25576000	3.85563200	0.46291200
H	2.25575600	-3.85562400	0.46291400
H	3.85929900	-5.57613400	1.05326000
H	6.27310100	-5.22369200	0.93124600
H	-6.27309700	-5.22368700	0.93125800
H	-3.85929400	-5.57612600	1.05327100
H	-2.25575300	-3.85561700	0.46291600
H	-2.25575900	3.85562700	0.46291100
H	-3.85930300	5.57613200	1.05326500
H	-6.27310400	5.22369000	0.93125200
H	10.90313000	1.12460600	1.45545000
H	10.51176700	3.52073500	1.74368600
H	8.39095900	4.58007200	1.15621400
H	8.39095500	-4.58007500	1.15621100
H	10.51176300	-3.52074100	1.74368600
H	10.90312800	-1.12461100	1.45545200
H	-10.90313000	-1.12461200	1.45544700
H	-10.51176400	-3.52074100	1.74368500
H	-8.39095400	-4.58007400	1.15621500
H	-8.39095700	4.58006800	1.15622000
H	-10.51176700	3.52073300	1.74368800
H	-10.90313200	1.12460400	1.45544700

***syn*-IV**

C	1.43175000	1.25228600	0.84945800
C	2.85198700	1.27325400	0.76564900
C	3.47161900	-0.00735500	0.85004200
C	2.84858200	-1.27374700	0.61750100
C	1.44081900	-1.20363600	0.38678000
C	0.72155100	0.01940900	0.64330100
C	0.68121400	-2.27501200	-0.17653400
C	-0.68120800	-2.27501100	-0.17654000
C	-1.44081600	-1.20363600	0.38677400
C	-0.72155000	0.01940900	0.64330100
C	-1.43174900	1.25228500	0.84946400
C	-0.68115800	2.41325300	1.20557800
C	0.68116100	2.41325400	1.20557400
C	3.69314400	2.48537200	0.49517600
C	5.11829200	2.34866100	0.54551600
C	5.62111900	1.12023500	0.95294400
N	4.82631400	-0.00103800	1.06576500
C	-2.84857900	-1.27375100	0.61749400
C	-3.47161800	-0.00736000	0.85004300
C	-2.85198700	1.27325200	0.76566000
C	5.61821100	-1.10069600	0.86704300
C	5.12621400	-2.33305400	0.48087100
C	3.70447500	-2.50768400	0.49286900
C	-3.70447300	-2.50768800	0.49285300
C	-5.12621200	-2.33305800	0.48085600
C	-5.61820900	-1.10070300	0.86703300
N	-4.82631300	-0.00104500	1.06576200
C	-5.62112100	1.12022700	0.95295100
C	-5.11829600	2.34865600	0.54553300
C	-3.69314800	2.48537000	0.49519400
C	6.10789800	-3.27586200	0.01586600
C	5.60435000	-4.56589000	-0.21522700
C	4.24952000	-4.83621500	-0.00220300
C	3.30682400	-3.84578700	0.32322500
C	3.23987900	3.71350300	-0.01377700
C	4.15402500	4.69972200	-0.41413900
C	5.53550100	4.49437100	-0.42990900

C	6.07794600	3.27095200	-0.00324300
C	-6.07795100	3.27095000	-0.00322000
C	-5.53550800	4.49437300	-0.42987600
C	-4.15403200	4.69972800	-0.41410200
C	-3.23988500	3.71350700	-0.01374700
C	-3.30682100	-3.84579000	0.32320200
C	-4.24951700	-4.83621700	-0.00223200
C	-5.60434700	-4.56589200	-0.21525300
C	-6.10789500	-3.27586500	0.01584500
C	-6.91874000	0.70865800	0.71834900
C	-6.91613100	-0.68624400	0.67831400
C	6.91873600	0.70866500	0.71833900
C	6.91613100	-0.68623600	0.67831600
C	7.84777100	1.47398700	0.01063500
C	8.95853600	0.76952600	-0.56919200
C	8.96574200	-0.74014100	-0.57669200
C	7.85004300	-1.44864300	-0.02060300
C	9.88534500	-1.54536300	-1.25958100
C	9.64815200	-2.92230500	-1.41570400
C	8.47315000	-3.54601200	-0.98378000
C	7.49498900	-2.80842700	-0.29256100
C	7.47956000	2.82595600	-0.27717400
C	8.44499400	3.56726400	-0.98307200
C	9.62195900	2.95316300	-1.42006900
C	9.86536100	1.57788300	-1.26449800
C	-7.85004200	-1.44864700	-0.02061200
C	-8.96574200	-0.74014400	-0.57669900
C	-8.95853800	0.76952400	-0.56918700
C	-7.84777300	1.47398200	0.01064600
C	-9.86536200	1.57788500	-1.26449000
C	-9.62196100	2.95316500	-1.42005300
C	-8.44499700	3.56726500	-0.98305100
C	-7.47956400	2.82595400	-0.27715600
C	-7.49498600	-2.80842900	-0.29257900
C	-8.47314600	-3.54601100	-0.98380300
C	-9.64814800	-2.92230300	-1.41572600
C	-9.88534200	-1.54536100	-1.25959500
H	1.19866600	-3.08215100	-0.67527200
H	-1.19865700	-3.08214400	-0.67529000
H	-1.21210400	3.29100100	1.55406200
H	1.21210700	3.29100400	1.55405100
H	6.25792900	-5.36685800	-0.54881400
H	3.89506500	-5.85532900	-0.13070800
H	2.27861400	-4.15241100	0.45779400
H	2.18457900	3.90189400	-0.16508600
H	3.76319000	5.64087300	-0.79148500
H	6.17650400	5.27219100	-0.83445500
H	-6.17651100	5.27219600	-0.83441600
H	-3.76320000	5.64088300	-0.79143900
H	-2.18458500	3.90190000	-0.16505100
H	-2.27861000	-4.15241200	0.45776700
H	-3.89506000	-5.85532900	-0.13074100
H	-6.25792600	-5.36685700	-0.54884500
H	10.77559400	-1.11651500	-1.71136600
H	10.38405500	-3.51580900	-1.95167100
H	8.31463600	-4.59313700	-1.22672400
H	8.27633500	4.61152700	-1.23027900
H	10.34986400	3.55008600	-1.96309700
H	10.74869800	1.14970700	-1.73017800
H	-10.74869700	1.14970900	-1.73017400
H	-10.34986500	3.55009100	-1.96308000
H	-8.27634000	4.61152900	-1.23025300
H	-8.31462900	-4.59313400	-1.22675200
H	-10.38405100	-3.51580300	-1.95169800
H	-10.77559000	-1.11651200	-1.71137900

***anti*-I**

C	1.42698200	-1.44387900	0.07275300
C	2.84791700	-1.43787200	0.13131500
C	3.41274900	-0.24802500	0.67822700
C	2.77691600	1.02762900	0.79872100
C	1.40820200	1.04164800	0.37015900
C	0.71227700	-0.19719500	0.12078800
C	0.65896800	2.23735400	0.17695000
C	-0.65897900	2.23735300	-0.17696600
C	-1.40820900	1.04164500	-0.37017800
C	-0.71228200	-0.19719600	-0.12080800
C	-1.42698500	-1.44388100	-0.07277400
C	-0.68141700	-2.65759100	-0.00616200
C	0.68141600	-2.65759000	0.00613700
C	3.74896200	-2.50492300	-0.41516600
C	5.15545800	-2.39788900	-0.16928900
C	5.57247700	-1.36594900	0.66024100
N	4.73621500	-0.33375200	1.03115800
C	-2.77692200	1.02762300	-0.79874100
C	-3.41275400	-0.24803100	-0.67824600
C	-2.84792000	-1.43787500	-0.13133300
C	5.51141500	0.76518500	1.28414700

C	5.02601100	2.05814600	1.27225900
C	3.60478400	2.22839100	1.19485300
C	-3.60479100	2.22838300	-1.19487200
C	-5.02601800	2.05814000	-1.27227100
C	-5.51142300	0.76517800	-1.28415800
N	-4.73622100	-0.33376000	-1.03117800
C	-5.57248300	-1.36595500	-0.66024600
C	-5.15545800	-2.39789200	0.16928600
C	-3.74896000	-2.50492400	0.41515400
C	6.02644700	3.09271200	1.25663000
C	5.51791100	4.39004900	1.42302700
C	4.14299200	4.58285200	1.58131900
C	3.19825000	3.54980500	1.45713700
C	3.38911000	-3.50256500	-1.33629300
C	4.37019800	-4.31191100	-1.92858600
C	5.73902000	-4.11868000	-1.72661100
C	6.19581800	-3.09988400	-0.87448200
C	-6.19581300	-3.09988400	0.87448800
C	-5.73900900	-4.11867700	1.72661700
C	-4.37018600	-4.31190900	1.92858300
C	-3.38910200	-3.50256400	1.33628200
C	-3.19825700	3.54979600	-1.45716000
C	-4.14299800	4.58284500	-1.58133700
C	-5.51791600	4.39004300	-1.42303400
C	-6.02645200	3.09270600	-1.25663300
C	-6.87486100	-0.90908200	-0.71673100
C	-6.83408400	0.42486700	-1.12350300
C	6.87485600	-0.90907800	0.71673800
C	6.83407700	0.42487200	1.12350400
C	7.89793200	-1.41156700	-0.09027500
C	9.04193400	-0.56361500	-0.28719600
C	9.00489400	0.86766500	0.19178100
C	7.81530300	1.36547200	0.81808400
C	9.96728400	1.84505200	-0.08925300
C	9.70638100	3.19999700	0.18089800
C	8.47409300	3.65751700	0.65897000
C	7.45077900	2.74164200	0.96330400
C	7.60413900	-2.59672800	-0.83534900
C	8.66084500	-3.07557000	-1.63163000
C	9.85790700	-2.35914500	-1.71652400
C	10.04125200	-1.10895000	-1.10161600
C	-7.81530600	1.36547000	-0.81807200
C	-9.00489100	0.86766400	-0.19175700
C	-9.04193000	-0.56361400	0.28722100
C	-7.89793000	-1.41156800	0.09029200
C	-10.04124300	-1.10894800	1.10164700
C	-9.85789500	-2.35914300	1.71655400
C	-8.66083400	-3.07556900	1.63165300
C	-7.60413300	-2.59672800	0.83536400
C	-7.45078100	2.74163900	-0.96329500
C	-8.47409000	3.65751500	-0.65895000
C	-9.70637400	3.19999800	-0.18086600
C	-9.96727600	1.84505400	0.08928800
H	1.14882500	3.19281500	0.26372500
H	-1.14883800	3.19281200	-0.26373900
H	-1.21065200	-3.60144400	-0.03929800
H	1.21065200	-3.60144300	0.03927100
H	6.18108600	5.25004400	1.44766900
H	3.77576200	5.58715400	1.77430600
H	2.15911100	3.80885400	1.60154000
H	2.36183800	-3.62784200	-1.65490800
H	4.05035100	-5.08003600	-2.62752200
H	6.44254700	-4.72802100	-2.28633300
H	-6.44253300	-4.72801600	2.28634700
H	-4.05033500	-5.08003100	2.62751900
H	-2.36182800	-3.62784000	1.65489000
H	-2.15911800	3.80884200	-1.60157000
H	-3.77576800	5.58714500	-1.77432700
H	-6.18109100	5.25003800	-1.44767100
H	10.91317400	1.58014500	-0.55368100
H	10.47672100	3.93058800	-0.05122200
H	8.31043400	4.72781800	0.74998600
H	8.55101900	-3.98322300	-2.21827000
H	10.65684100	-2.75247100	-2.33945700
H	10.95516600	-0.55767100	-1.30504100
H	-10.95515500	-0.55766900	1.30507800
H	-10.65682500	-2.75247000	2.33949200
H	-8.55100400	-3.98322300	2.21829100
H	-8.31043000	4.72781600	-0.74996800
H	-10.47671100	3.93059000	0.05126200
H	-10.91316100	1.58014800	0.55372600

***anti*-II**

C	1.44527200	-1.38842200	0.17866500
C	2.86800200	-1.39717800	0.25513400
C	3.44434500	-0.15060000	0.63410500
C	2.82968600	1.13681200	0.53478500
C	1.46968400	1.10746500	0.10034700

C	0.74212300	-0.13825100	0.08124100	C	6.86474000	0.55037200	1.04023600
C	0.78478300	2.25481700	-0.40310000	C	7.92588900	-1.45948900	0.13825200
C	-0.55737900	2.25114400	-0.63377000	C	9.08563300	-0.66428700	-0.15937300
C	-1.37057600	1.10761300	-0.36728200	C	9.05833100	0.82333100	0.09470200
C	-0.69242300	-0.13311100	-0.08814700	C	7.86337000	1.42341400	0.61190300
C	-1.42966300	-1.36674500	0.02861900	C	10.03946800	1.73508900	-0.31326000
C	-0.68413500	-2.57576300	0.16807800	C	9.79219900	3.11806100	-0.26159700
C	0.67785000	-2.58848400	0.21696500	C	8.55654100	3.65639800	0.11161300
C	3.76688700	-2.54012300	-0.11703800	C	7.51475700	2.80944000	0.53124900
C	5.17002300	-2.41501800	0.14612000	C	7.62954900	-2.73886200	-0.42814700
C	5.58763600	-1.27792800	0.82596400	C	8.69514000	-3.34473900	-1.11923100
N	4.75909800	-0.19305400	1.02274200	C	9.90447900	-2.66385900	-1.28338400
C	-2.78581300	1.13922900	-0.52029900	C	10.09339800	-1.33740900	-0.85976200
C	-3.41571900	-0.13918000	-0.53395500	C	-7.84350500	1.42531500	-0.76987100
C	-2.85224400	-1.36831200	-0.08568000	C	-9.06914900	0.82294300	-0.32865300
C	5.54384400	0.92515300	1.12224500	C	-9.11071400	-0.66001900	-0.06436100
C	5.07798500	2.20549400	0.88926100	C	-7.92437100	-1.44395600	-0.25958500
C	3.66379000	2.37399900	0.73756300	C	-10.16346200	-1.33198100	0.56786900
C	-3.64703400	2.37091900	-0.55796900	C	-9.99371200	-2.64781500	1.02965300
C	-5.03897000	2.21953900	-0.86641300	C	-8.76677900	-3.31437200	0.97386700
C	-5.48855500	0.93967100	-1.16915000	C	-7.65829100	-2.70743100	0.35520400
N	-4.71659900	-0.17799100	-0.95918200	C	-7.50334000	2.80722100	-0.62784800
C	-5.55013900	-1.25665700	-0.78173500	C	-8.57144700	3.64982500	-0.26873600
C	-5.16706900	-2.37491100	-0.05001600	C	-9.83229700	3.11125200	0.00158400
C	-3.78200800	-2.48516600	0.30524200	C	-10.07793100	1.72820000	0.02123300
C	6.08864600	3.21559400	0.72675500	H	1.35759800	3.12705200	-0.68514400
C	5.58832500	4.52549200	0.65180000	H	-1.00914300	3.11648900	-1.10073400
C	4.21098700	4.75352500	0.72198500	H	-1.20770600	-3.52089100	0.16976600
C	3.25826800	3.72034200	0.74407900	H	1.18680800	-3.53522700	0.34117000
C	3.41560600	-3.65246700	-0.89966200	H	6.26049600	5.37272500	0.54996100
C	4.39706900	-4.55022200	-1.34576800	H	3.85191800	5.77910800	0.72065400
C	5.76285500	-4.34875200	-1.13582300	H	2.21329800	3.99624600	0.78647300
C	6.21469200	-3.22409200	-0.42620700	H	2.39639300	-3.80753800	-1.22939600
C	-6.24343400	-3.17535300	0.47251600	H	4.08137700	-5.40448800	-1.93857000
C	-5.83865600	-4.26987500	1.25364500	H	6.47007400	-5.04011600	-1.58443500
C	-4.49228900	-4.44537100	1.57535800	H	-6.57235500	-4.95140500	1.67361600
C	-3.48691800	-3.55804300	1.16285400	H	-4.21461000	-5.26859000	2.22806800
C	-3.28166100	3.65945300	-0.13434600	H	-2.49774900	-3.68963600	1.58035500
C	-4.23298000	4.68874300	-0.06857800	H	-2.27919200	3.87367500	0.21131700
C	-5.59116200	4.48449800	-0.31607600	H	-3.90357200	5.67325600	0.25250400
C	-6.06425100	3.20951100	-0.66708100	H	-6.28189200	5.30923300	-0.16784100
C	-6.85333900	-0.81825900	-0.90181800	H	10.99037700	1.39117900	-0.71075300
C	-6.81456000	0.55855500	-1.14529900	H	10.57710000	3.79589100	-0.58647000
C	6.89493000	-0.83082500	0.84001000	H	8.40544600	4.72943600	0.03202600

H	8.58373500	-4.32906900	-1.56498800
H	10.71025200	-3.15603100	-1.82146600
H	11.01896800	-0.83335800	-1.12373000
H	-11.11205600	-0.83455900	0.74950000
H	-10.83340400	-3.14114500	1.51188900
H	-8.67851600	-4.28891400	1.44544100
H	-8.42582000	4.72099700	-0.16238200
H	-10.63939800	3.78571400	0.27490600
H	-11.05077300	1.37666400	0.35328700

***anti*-IV**

C	1.45859300	1.23984600	-0.00126700
C	2.82815200	1.29902800	-0.41298300
C	3.41075500	0.02715700	-0.71356200
C	2.79559000	-1.24900200	-0.56327800
C	1.37876400	-1.21616800	-0.45838200
C	0.71163000	0.00979000	-0.12377700
C	0.57316000	-2.34963400	-0.77544700
C	-0.77585200	-2.34925300	-0.58652100
C	-1.45858800	-1.23983400	0.00121900
C	-0.71162600	-0.00977800	0.12372800
C	-1.37876100	1.21618000	0.45833200
C	-0.57315800	2.34964900	0.77538800
C	0.77585400	2.34926700	0.58646300
C	3.70560300	2.52775800	-0.40628500
C	5.11890600	2.33548400	-0.54654700
C	5.55134700	1.09760600	-0.98100600
N	4.73023800	0.00774700	-1.09002100
C	-2.82814400	-1.29902100	0.41294800
C	-3.41074700	-0.02715000	0.71353400
C	-2.79558700	1.24901000	0.56324000
C	5.52148900	-1.12163000	-1.07428900
C	5.05713800	-2.34930600	-0.62341600
C	3.64590300	-2.47462700	-0.41630400
C	-3.70559300	-2.52775300	0.40625800
C	-5.11889400	-2.33548200	0.54654100
C	-5.55133300	-1.09760600	0.98100700
N	-4.73022500	-0.00774400	1.09001000
C	-5.52147900	1.12163100	1.07428900
C	-5.05713800	2.34930800	0.62340600
C	-3.64590600	2.47463100	0.41627300
C	6.06307200	-3.28577300	-0.19584100
C	5.55879600	-4.51102900	0.27056700
C	4.18131300	-4.70739600	0.39906400
C	3.23743200	-3.70858400	0.11575400
C	3.35005700	3.87615700	-0.22033400
C	4.33580300	4.85585200	-0.00940900
C	5.70123300	4.56783400	0.06701400
C	6.15859500	3.26800700	-0.20119500
C	-6.06308000	3.28577100	0.19584300
C	-5.55881300	4.51102700	-0.27057600
C	-4.18133300	4.70739700	-0.39909500
C	-3.23744500	3.70858800	-0.11579400
C	-3.35004500	-3.87615000	0.22029500
C	-4.33579200	-4.85584700	0.00938300
C	-5.70122400	-4.56783300	-0.06701600
C	-6.15858600	-3.26800800	0.20120300
C	-6.84166500	0.72457800	0.98923900
C	-6.85801000	-0.66980200	0.94463400
C	6.85802200	0.66979900	-0.94461400
C	6.84167400	-0.72458100	-0.98922000
C	7.87329200	1.42045300	-0.35535800
C	9.03580600	0.69822200	0.07207000
C	9.01193700	-0.81150100	0.06113500
C	7.83702700	-1.50295700	-0.39468500
C	9.98142900	-1.63148900	0.65029400
C	9.74323200	-3.00510100	0.82707200
C	8.51998600	-3.60675800	0.51865400
C	7.49011500	-2.85338500	-0.07440000
C	7.56533000	2.78336400	-0.04516200
C	8.62142700	3.50851800	0.53576300
C	9.82949400	2.87060000	0.83574800
C	10.03329200	1.49169000	0.65166800
C	-7.87328500	-1.42045900	0.35539200
C	-9.03580800	-0.69823200	-0.07202000
C	-9.01194300	0.81149100	-0.06108700
C	-7.83702800	1.50295100	0.39471600
C	-9.98144500	1.63147600	-0.65023400
C	-9.74325400	3.00508800	-0.82701700
C	-8.52000500	3.60674900	-0.51861700
C	-7.49012400	2.85338000	0.07442400
C	-7.56532400	-2.78336900	0.04519200
C	-8.62142800	-3.50852700	-0.53571800
C	-9.82950100	-2.87061200	-0.83568600
C	-10.03330000	-1.49170200	-0.65160300
H	1.04211100	-3.20208500	-1.25351400
H	-1.34601800	-3.19357000	-0.94306700
H	-1.04211000	3.20210400	1.25344700

H	1.34601700	3.19358800	0.94300300
H	6.23292100	-5.29944400	0.59238200
H	3.82571300	-5.65273700	0.80003400
H	2.20145800	-3.89212400	0.37232900
H	2.32043500	4.20297200	-0.25979000
H	4.01039200	5.88204700	0.13812000
H	6.39746300	5.36273000	0.31888400
H	-6.23294500	5.29943800	-0.59238500
H	-3.82574100	5.65273700	-0.80007300
H	-2.20147500	3.89212900	-0.37238500
H	-2.32042200	-4.20296100	0.25973200
H	-4.01038000	-5.88204000	-0.13815500
H	-6.39745600	-5.36273000	-0.31887800
H	10.91447900	-1.21349900	1.01796400
H	10.52022400	-3.61090900	1.28568500
H	8.36898500	-4.65043500	0.77955100
H	8.50136400	4.55630600	0.79711100
H	10.62595800	3.45433400	1.28957700
H	10.96297300	1.05204900	1.00236500
H	-10.91449900	1.21348300	-1.01789000
H	-10.52025400	3.61089400	-1.28562000
H	-8.36900900	4.65042600	-0.77951800
H	-8.50136500	-4.55631300	-0.79706800
H	-10.62597000	-3.45434800	-1.28950200
H	-10.96298700	-1.05206400	-1.00228600

***anti*-V**

C	-1.44362100	-1.25155200	-0.05700400
C	-2.86513300	-1.27078700	-0.17663700
C	-3.45752700	-0.00000100	-0.42636500
C	-2.86513500	1.27078900	-0.17665200
C	-1.44362300	1.25155500	-0.05701600
C	-0.72360400	0.00000200	-0.03398200
C	-0.67953800	2.45419700	-0.03434700
C	0.67953900	2.45419700	0.03441800
C	1.44362600	1.25155700	0.05707700
C	0.72360600	0.00000300	0.03404600
C	1.44362400	-1.25155000	0.05706900
C	0.67954000	-2.45419200	0.03441800
C	-0.67953700	-2.45419400	-0.03433200
C	-3.77407400	-2.45820900	0.01108100
C	-5.14989800	-2.33066900	-0.37503200
C	-5.54837300	-1.11616200	-0.92342400
N	-4.74714800	-0.00000600	-0.88341500
C	2.86513900	1.27079100	0.17670100
C	3.45753500	0.00000100	0.42640700
C	2.86513700	-1.27078500	0.17668600
C	-5.54837400	1.11614800	-0.92344300
C	-5.14990200	2.33066400	-0.37506600
C	-3.77407900	2.45821200	0.01104800
C	3.77408100	2.45821300	-0.01101300
C	5.14991000	2.33066400	0.37507700
C	5.54839100	1.11615000	0.92345300
N	4.74716200	-0.00000400	0.88343400
C	5.54838800	-1.11616100	0.92343000
C	5.14990200	-2.33066700	0.37504100
C	3.77407300	-2.45820500	-0.01104700
C	-6.21515800	3.23620300	-0.03176200
C	-5.80174500	4.43008000	0.58120400
C	-4.46425000	4.61334800	0.93112000
C	-3.47675900	3.64247000	0.70597200
C	-3.47675000	-3.64245700	0.70602000
C	-4.46423900	-4.61333300	0.93118300
C	-5.80173500	-4.43007200	0.58126900
C	-6.21515200	-3.23620400	-0.03171300
C	6.21515000	-3.23620300	0.03170300
C	5.80172300	-4.43006700	-0.58127700
C	4.46422000	-4.61332800	-0.93116700
C	3.47673500	-3.64245400	-0.70598200
C	3.47675000	3.64247500	-0.70592900
C	4.46423900	4.61335000	-0.93109300
C	5.80174000	4.43007900	-0.58120300
C	6.21516000	3.23620200	0.03175600
C	6.86125100	-0.69966800	1.01255100
C	6.86125300	0.69965100	1.01256400
C	-6.86123500	-0.69966900	-1.01256800
C	-6.86123600	0.69965200	-1.01257800
C	-7.93026700	-1.45654000	-0.52672500
C	-9.14905700	-0.75317500	-0.24493300
C	-9.14905900	0.75316600	-0.24494600
C	-7.93027000	1.45652800	-0.52674800
C	-10.19833900	1.55419200	0.22056400
C	-9.99819800	2.92463000	0.45572300
C	-8.74705500	3.53499800	0.33553100
C	-7.64070200	2.79799800	-0.12599400
C	-7.64069600	-2.79800300	-0.12594800
C	-8.74704700	-3.53499600	0.33559200
C	-9.99819000	-2.92462800	0.45577700

C	-10.19833500	-1.55419500	0.22059400
C	7.93028000	1.45652700	0.52671500
C	9.14906100	0.75316400	0.24488800
C	9.14905900	-0.75317700	0.24487600
C	7.93027500	-1.45654000	0.52668900
C	10.19832900	-1.55419800	-0.22067000
C	9.99818100	-2.92463100	-0.45584700
C	8.74703900	-3.53499700	-0.33564000
C	7.64069700	-2.79800300	0.12591800
C	7.64070600	2.79799600	0.12596400
C	8.74704900	3.53499400	-0.33558500
C	9.99818900	2.92462400	-0.45580500
C	10.19833300	1.55418700	-0.22064800
H	-1.18452300	3.40439000	-0.11741400
H	1.18452200	3.40439000	0.11749200
H	1.18452900	-3.40438400	0.11748900
H	-1.18452400	-3.40438700	-0.11738400
H	-6.52616300	5.19082700	0.85608400
H	-4.18191100	5.51960200	1.46010600
H	-2.50331200	3.80899000	1.14625400
H	-2.50330100	-3.80896900	1.14630100
H	-4.18189700	-5.51958000	1.46018200
H	-6.52615100	-5.19081700	0.85615900
H	6.52613500	-5.19080800	-0.85619000
H	4.18186900	-5.51957300	-1.46016400
H	2.50327600	-3.80896600	-1.14624200
H	2.50329700	3.80899800	-1.14619400
H	4.18189100	5.51960500	-1.46007400
H	6.52615500	5.19082300	-0.85609800
H	-11.16933000	1.12305200	0.44759700
H	-10.83585500	3.51818300	0.81215300
H	-8.63986700	4.57331700	0.63591000
H	-8.63985600	-4.57331000	0.63598900
H	-10.83584500	-3.51817600	0.81222100
H	-11.16932500	-1.12305300	0.44762400
H	11.16931500	-1.12305500	-0.44771800
H	10.83582900	-3.51817900	-0.81230500
H	8.63984300	-4.57331300	-0.63602900
H	8.63985600	4.57331400	-0.63596000
H	10.83583800	3.51817500	-0.81225600
H	11.16931700	1.12304400	-0.44770600

TS$_{hel}$

C	1.44149400	-1.23143400	-0.69111200
C	2.86097600	-1.28025100	-0.64002000
C	3.50885000	-0.02764100	-0.84295500
C	2.91922600	1.26620200	-0.70660900
C	1.51663800	1.24495200	-0.42088000
C	0.75815300	0.02518300	-0.53789500
C	0.79863100	2.38494200	0.03611300
C	-0.55895000	2.40169800	0.14836100
C	-1.38655800	1.28811800	-0.18557900
C	-0.68711300	0.05050400	-0.45976600
C	-1.41451600	-1.18033400	-0.62696000
C	-0.69038100	-2.36340400	-0.96926000
C	0.66902800	-2.39495100	-0.97868000
C	3.67624900	-2.48997400	-0.29358900
C	5.10271000	-2.39422000	-0.37851900
C	5.62664900	-1.21702800	-0.89544500
N	4.85728100	-0.08882900	-1.08812800
C	-2.82092600	1.35629900	-0.26186200
C	-3.44003900	0.10779800	-0.58819600
C	-2.83161200	-1.18305900	-0.56329500
C	5.67945100	1.00229600	-0.99650700
C	5.22464900	2.27534500	-0.71090100
C	3.80710800	2.48609700	-0.71698200
C	-3.72157700	2.54734200	0.03393100
C	-5.14009000	2.38708300	-0.14993500
C	-5.58830500	1.20494400	-0.71605200
N	-4.77502000	0.11746700	-0.89425300
C	-5.56759700	-1.00686200	-0.96401900
C	-5.10464900	-2.26554800	-0.60371200
C	-3.69377700	-2.40341900	-0.41310600
C	6.23930200	3.22900000	-0.34901000
C	5.77513700	4.54865300	-0.23229800
C	4.42549400	4.83568500	-0.45498300
C	3.45131000	3.84629900	-0.67336200
C	3.20163500	-3.66247100	0.31677500
C	4.09898600	-4.63598300	0.78132200
C	5.48514400	-4.46327400	0.76159700
C	6.04946400	-3.29247400	0.22866000
C	-6.10988600	-3.22600000	-0.23421900
C	-5.60735600	-4.47106700	0.17829700
C	-4.23206500	-4.66567300	0.32067600
C	-3.28940600	-3.65379600	0.08323000
C	-3.38746700	3.80101900	0.58097300
C	-4.37003100	4.73509800	0.94251500
C	-5.73665800	4.47966400	0.85742100

C	-6.18321100	3.25887100	0.33424700
C	-6.88542900	-0.61393000	-0.85182900
C	-6.89814100	0.77479600	-0.70510900
C	6.93826800	-0.82114300	-0.71903900
C	6.97051600	0.57186200	-0.79719700
C	7.86127700	-1.54809500	0.03587600
C	8.99846200	-0.82582700	0.53712800
C	9.04276900	0.67844300	0.41637400
C	7.93563800	1.36609600	-0.18108600
C	9.99296300	1.51432600	1.01542300
C	9.79242100	2.90526300	1.05747800
C	8.62643400	3.52064200	0.59053800
C	7.61901800	2.75274800	-0.02117900
C	7.46572800	-2.86118400	0.44282600
C	8.42478000	-3.56461400	1.19477000
C	9.62318700	-2.94576500	1.56102900
C	9.89692000	-1.59521100	1.28555400
C	-7.91420000	1.46511100	-0.04594100
C	-9.08267500	0.71350800	0.31038700
C	-9.06437700	-0.78733800	0.17141100
C	-7.88357300	-1.43713700	-0.32413700
C	-10.04139200	-1.65064700	0.68099800
C	-9.80362200	-3.03337800	0.75301600
C	-8.57386400	-3.60612400	0.41758800
C	-7.53850200	-2.80709200	-0.10134000
C	-7.59789400	2.78178300	0.40881100
C	-8.65174400	3.45545600	1.05379700
C	-9.86869200	2.80508000	1.27725500
C	-10.07926500	1.45255600	0.95830700
H	1.34199800	3.26120400	0.35491900
H	-0.99591000	3.31281900	0.50932500
H	-1.24118100	-3.23529800	-1.29715400
H	1.18064100	-3.29544500	-1.29772400
H	6.45525400	5.35839800	0.01612400
H	4.10050400	5.87201300	-0.42137000
H	2.43234800	4.17200700	-0.82994600
H	2.14404900	-3.81184500	0.49513800
H	3.69279800	-5.53465700	1.23767800
H	6.11433000	-5.22123200	1.21894300
H	-6.28247000	-5.27514200	0.45593300
H	-3.87680300	-5.62291400	0.69259000
H	-2.25771200	-3.84423300	0.35014200
H	-2.37308300	4.10309500	0.77554900
H	-4.03880800	5.68441000	1.35471900
H	-6.44031300	5.21979800	1.22631900
H	10.87946300	1.10207900	1.48944600
H	10.55131500	3.52256900	1.53083900
H	8.49748300	4.58824400	0.74608900
H	8.23488600	-4.57956500	1.53225000
H	10.34542500	-3.51285100	2.14225700
H	10.79776700	-1.15150200	1.70033300
H	-10.97982300	-1.26258700	1.06711800
H	-10.58604200	-3.67451500	1.15025700
H	-8.42348200	-4.66683200	0.59726300
H	-8.52620400	4.47062400	1.41935700
H	-10.66474700	3.34908700	1.77863200
H	-11.01228000	0.98533900	1.26093200

TS_{bowl}

C	-1.46100800	-1.31432400	-0.21642200
C	-2.88777900	-1.33700700	-0.23246900
C	-3.49910200	-0.10904700	-0.61230700
C	-2.88824200	1.17766800	-0.59961700
C	-1.46466000	1.16622200	-0.55407100
C	-0.74353400	-0.07200600	-0.37282400
C	-0.70485500	2.34515100	-0.82093900
C	0.65004000	2.36914600	-0.70537500
C	1.39865600	1.20636900	-0.35684800
C	0.70394200	-0.06458400	-0.33380900
C	1.42745800	-1.32009700	-0.27723300
C	0.66340200	-2.51714700	-0.16805700
C	-0.69760700	-2.51520800	-0.13392100
C	-3.77407900	-2.47740700	0.19256900
C	-5.17966900	-2.38567800	-0.07855900
C	-5.62601400	-1.25724200	-0.75747000
N	-4.82337000	-0.15885700	-0.95492900
C	2.79987900	1.26186300	-0.12048800
C	3.41997000	-0.02855600	-0.17590800
C	2.85506400	-1.33612500	-0.32537100
C	-5.62463200	0.95036100	-1.08434200
C	-5.17760200	2.22762900	-0.76533000
C	-3.77226400	2.39334700	-0.53256000
C	3.64300300	2.51659300	0.14866300
C	5.07197700	2.38260300	0.17437000
C	5.53220800	1.10817200	0.02159600
N	4.76430100	-0.00231800	-0.07553000
C	5.57773400	-1.08276300	-0.04196700
C	5.19243800	-2.37257400	-0.25964300

C	3.78305900	-2.55929800	-0.47186900
C	-6.20509700	3.19120400	-0.46986700
C	-5.73358300	4.46039200	-0.09684300
C	-4.36754000	4.67877900	0.08491800
C	-3.40566000	3.66993700	-0.07478500
C	-3.41112700	-3.56032400	1.01074100
C	-4.37415200	-4.47574600	1.46091400
C	-5.73949300	-4.32107900	1.21935700
C	-6.20869200	-3.21837900	0.48743900
C	6.30278100	-3.29906900	-0.34662900
C	5.88014200	-4.59495500	-0.68408600
C	4.52310800	-4.84407000	-0.94301800
C	3.49628500	-3.87942400	-0.86647600
C	3.23678900	3.82508300	0.46642000
C	4.19881100	4.82921500	0.70612700
C	5.58924100	4.62966300	0.68931200
C	6.11449300	3.35049400	0.44055400
C	6.85824400	-0.64687200	0.07062200
C	6.82903200	0.72053700	0.12030500
C	-6.94131700	-0.83952800	-0.78560500
C	-6.94034000	0.54429800	-0.99079300
C	-7.96063300	-1.50209500	-0.09761000
C	-9.14649500	-0.74667700	0.19031600
C	-9.14567700	0.74342800	-0.03197200
C	-7.95875000	1.38042300	-0.52702800
C	-10.14636700	1.61938000	0.40487600
C	-9.92490000	3.00655900	0.41347400
C	-8.69105400	3.57526800	0.08651000
C	-7.63293300	2.76296800	-0.36115600
C	-7.63622300	-2.77591800	0.46532400
C	-8.69498200	-3.42071700	1.13103900
C	-9.92809700	-2.77994100	1.27766200
C	-10.14789400	-1.45549200	0.86429900
C	7.90808600	1.53576900	0.32663500
C	9.17056500	0.84584600	0.39151000
C	9.21426100	-0.68628800	0.21094300
C	7.98543200	-1.42129800	0.05846400
C	10.33435300	-1.52674600	0.16420300
C	10.16856800	-2.91832900	-0.02479600
C	8.93110800	-3.56666500	-0.18501500
C	7.73880700	-2.81769600	-0.15134500
C	7.57906700	2.92152900	0.49329200
C	8.71997600	3.71161700	0.73328200
C	9.98897500	3.10857600	0.78962400
C	10.23463800	1.72495400	0.63247700
H	-1.21436800	3.22458900	-1.19073100
H	1.18707100	3.27268800	-0.96468800
H	1.17709600	-3.46192400	-0.07541600
H	-1.21050600	-3.46551800	-0.10780600
H	-6.42803100	5.26602200	0.12199700
H	-4.03618400	5.65503900	0.42835600
H	-2.39025000	3.88838000	0.22740500
H	-2.39814500	-3.67972900	1.37051000
H	-4.04411900	-5.30560000	2.07996600
H	-6.43512000	-5.02536500	1.66568300
H	6.59145200	-5.41061100	-0.78578600
H	4.23980400	-5.84914200	-1.24402600
H	2.50377100	-4.19113700	-1.16559300
H	2.19216000	4.09154500	0.57365400
H	3.83265800	5.82536800	0.93998500
H	6.24082100	5.47297400	0.90374200
H	-11.09164300	1.24045100	0.78315600
H	-10.72511000	3.65798100	0.75452100
H	-8.55653000	4.64514800	0.21743100
H	-8.56158600	-4.40579500	1.56886100
H	-10.72886200	-3.30235100	1.79424400
H	-11.09240900	-0.98126800	1.11576800
H	11.34367400	-1.13456900	0.26389300
H	11.06604900	-3.53070600	-0.05535600
H	8.92322300	-4.64352900	-0.33528600
H	8.64721600	4.78633400	0.88153700
H	10.84496700	3.75197700	0.97598400
H	11.25914300	1.36896100	0.71071400

4b–C_{120}

C	-0.79180000	2.33463400	0.80970300
C	-0.79084200	2.20452200	-0.78323700
C	-1.50070700	0.97894300	-1.33855300
C	-1.94003100	-0.05859300	-0.54367700
C	-1.94321800	0.06206400	0.93636200
C	-1.50566100	1.21530700	1.55409000
C	-1.49689500	3.32660900	-1.52938500
C	-1.92863300	4.48258400	-0.91466700
C	-1.93163200	4.60319400	0.56386600
C	-1.50130500	3.56322900	1.36055000
C	-2.25376900	3.20887800	2.53339300
C	-2.25729300	1.76040500	2.65204600
C	-3.10566100	5.36296900	0.93533000

C	-3.82644800	5.71405300	-0.27559900	C	-7.57418900	3.57738800	1.32106400
C	-3.10035900	5.17162000	-1.41028000	C	-8.02524000	2.34798200	0.69057700
C	-3.80443300	4.67333700	-2.51278900	C	-8.02175600	2.23402300	-0.70134200
C	-3.36512300	3.45015900	-3.14005400	C	0.79086700	2.20452200	-0.78323700
C	-2.24263800	2.78669800	-2.63335100	C	0.79182600	2.33463100	0.80970300
C	-2.24602800	1.33827000	-2.51544200	C	1.50133300	3.56322300	1.36055100
C	-3.37310200	0.60932000	-2.90845700	C	1.93166200	4.60318900	0.56386900
C	-3.82048900	-0.48988600	-2.08811100	C	1.92866200	4.48258200	-0.91466400
C	-3.11828100	-0.80850100	-0.92077900	C	1.49692100	3.32660900	-1.52938400
C	-3.84773700	-1.15381500	0.28575500	C	1.50568500	1.21530100	1.55408800
C	-3.12300700	-0.61812700	1.42514000	C	1.94324000	0.06205900	0.93635700
C	-3.83120500	-0.11529200	2.52211200	C	1.94005200	-0.05859600	-0.54368200
C	-3.38732900	1.10400400	3.15245500	C	1.50072900	0.97894200	-1.33855600
C	-3.81488500	5.05013300	2.10077000	C	2.24604900	1.33827100	-2.51544500
C	-3.37881600	3.94487500	2.91981600	C	2.24266300	2.78669900	-2.63335200
C	-4.55807000	3.25994000	3.42749600	C	3.11830100	-0.80850400	-0.92078600
C	-4.56256400	1.87048800	3.54081800	C	3.84775700	-1.15382300	0.28574700
C	-5.22061000	5.73735300	-0.28076200	C	3.12302800	-0.61813600	1.42513300
C	-5.25641300	4.68381500	-2.51380700	C	3.83122800	-0.11530400	2.52210700
C	-5.71409300	3.45685700	-3.14687300	C	3.38735400	1.10399300	3.15245100
C	-4.54220800	2.69144800	-3.53567800	C	2.25731900	1.76039600	2.65204400
C	-4.54653400	1.30181900	-3.42211800	C	2.25379800	3.20887000	2.53339300
C	-5.72357600	0.61638300	-2.91518900	C	3.37884600	3.94486400	2.91981700
C	-5.27126900	-0.49045300	-2.08809700	C	3.81491600	5.05012300	2.10077300
C	-5.96766400	-0.81918200	-0.92586400	C	3.10569200	5.36296200	0.93533400
C	-5.24171700	-1.16850900	0.28402100	C	3.82647900	5.71404600	-0.27559500
C	-5.95099400	5.20430600	-1.42025900	C	3.10038800	5.17161700	-1.41027700
C	-5.95628500	5.39597700	0.92675100	C	3.80446100	4.67333500	-2.51278700
C	-5.26684800	5.06018300	2.09338100	C	3.36514800	3.45015900	-3.14005400
C	-5.72778700	3.95185600	2.91474400	C	3.82050900	-0.48989000	-2.08811800
C	-5.73775600	1.11112700	3.14775600	C	3.37312300	0.60931900	-2.90846200
C	-5.28373200	-0.11777000	2.51726400	C	4.54655600	1.30181700	-3.42212300
C	-5.97505000	-0.62933000	1.41930200	C	4.54223200	2.69144700	-3.53568000
C	-6.84540200	2.79966500	-2.65816000	C	5.24173700	-1.16851800	0.28401200
C	-6.85169500	1.35005900	-2.54127400	C	5.28375500	-0.11778400	2.51725700
C	-7.14195600	-0.06206300	-0.54079200	C	5.73778100	1.11111200	3.14775100
C	-7.57775300	1.00161200	-1.33132600	C	4.56259000	1.87047300	3.54081500
C	-7.56740100	3.34487900	-1.52125800	C	4.55809900	3.25992600	3.42749500
C	-7.14625300	0.05545900	0.91006500	C	5.72781700	3.95184100	2.91474400
C	-7.12870400	4.52467800	-0.91634400	C	5.26688000	5.06017100	2.09338300
C	-7.58478700	1.23443800	1.51467100	C	5.95631600	5.39596500	0.92675300
C	-7.13215900	4.64339600	0.53463200	C	5.22064100	5.73734400	-0.28075900
C	-6.86404000	1.77477000	2.65454100	C	5.97507200	-0.62934300	1.41929300
C	-6.85741300	3.22409500	2.53452500	C	5.96768400	-0.81919000	-0.92587300

C	5.27128800	-0.49045800	-2.08810600	C	5.63534200	-4.43545300	1.10638900
C	5.72359600	0.61637900	-2.91519500	N	4.84611300	-4.57418400	-0.01313100
C	5.71411800	3.45685200	-3.14687400	C	5.63940700	-4.42833100	-1.12923500
C	5.25644100	4.68381000	-2.51380500	C	5.12972200	-3.98847200	-2.34708000
C	5.95102300	5.20429900	-1.42025600	C	3.70462200	-3.91394700	-2.47728900
C	6.86406600	1.77475300	2.65453600	C	-6.07624300	-3.44924600	3.24569900
C	6.85744100	3.22407800	2.53452300	C	-5.53292300	-2.98087000	4.45285600
C	7.13218800	4.64338300	0.53463200	C	-4.15117900	-2.96181600	4.64345300
C	7.57421800	3.57737200	1.32106200	C	-3.24319800	-3.37755800	3.65952000
C	7.58481100	1.23442200	1.51466500	C	-3.25411500	-3.32091100	-3.66796600
C	7.12873200	4.52466700	-0.91634400	C	-4.16421700	-2.88931700	-4.64318000
C	7.14627500	0.05544500	0.91005700	C	-5.54568600	-2.91940300	-4.45261000
C	7.56742800	3.34486900	-1.52126000	C	-6.08669000	-3.41419400	-3.25459100
C	7.14197700	-0.06207400	-0.54080100	C	6.08665300	-3.41421400	-3.25458600
C	6.84542600	2.79965800	-2.65816200	C	5.54565200	-2.91941100	-4.45260100
C	6.85171700	1.35005200	-2.54128000	C	4.16418400	-2.88931700	-4.64317200
C	7.57777500	1.00160200	-1.33133200	C	3.25407800	-3.32091400	-3.66796300
C	8.02178100	2.23401100	-0.70134700	C	3.24315600	-3.37756400	3.65952100
C	8.02526600	2.34796700	0.69057200	C	4.15114000	-2.96182500	4.64345400
C	-1.43819800	-4.31506500	-1.26471500	C	5.53288400	-2.98088700	4.45285700
C	-2.86024300	-4.24485900	-1.28337900	C	6.07620200	-3.44926800	3.24570100
C	-3.48946100	-4.38400400	-0.01338400	C	6.93929700	-4.21625500	-0.70947800
C	-2.85814400	-4.26153400	1.25720900	C	6.93656200	-4.22031900	0.69264500
C	-1.43658400	-4.33545000	1.23495500	C	-6.93934000	-4.21622500	-0.70948000
C	-0.72235500	-4.32463000	-0.01510100	C	-6.93660600	-4.22028800	0.69264200
C	-0.68032800	-4.56977100	2.42286000	C	-7.86017800	-3.48015200	-1.46361100
C	0.68028200	-4.56977200	2.42286000	C	-8.95818200	-2.89127400	-0.75148400
C	1.43653800	-4.33545200	1.23495600	C	-8.95283100	-2.89301900	0.75278100
C	0.72231000	-4.32463200	-0.01510100	C	-7.85236900	-3.48809800	1.45590600
C	1.43815400	-4.31506800	-1.26471500	C	-9.83089300	-2.14576400	1.54568800
C	0.68044700	-4.52579100	-2.45655100	C	-9.57312800	-1.95590000	2.91277100
C	-0.68049200	-4.52579000	-2.45655100	C	-8.40689200	-2.41529400	3.52737400
C	-3.70466300	-3.91393900	-2.47729100	C	-7.47168100	-3.17270500	2.79873300
C	-5.12976300	-3.98845500	-2.34708300	C	-7.48385600	-3.15105400	-2.80407400
C	-5.63945200	-4.42831100	-1.12923700	C	-8.42209300	-2.38774500	-3.52236600
N	-4.84615900	-4.57416900	-0.01313200	C	-9.58928800	-1.93879700	-2.90094800
C	2.85809800	-4.26154000	1.25721000	C	-9.84218900	-2.14139400	-1.53517100
C	3.48941600	-4.38401300	-0.01338400	C	7.85233000	-3.48813600	1.45591000
C	2.86019900	-4.24486700	-1.28337800	C	8.95279800	-2.89306600	0.75278500
C	-5.63538800	-4.43543200	1.10638700	C	8.95814900	-2.89132100	-0.75148000
C	-5.12282300	-4.01175500	2.32819800	C	7.86014000	-3.48018900	-1.46360700
C	-3.69731500	-3.94512300	2.45804400	C	9.84216200	-2.14144800	-1.53516600
C	3.69727100	-3.94513200	2.45804500	C	9.58926300	-1.93884900	-2.90094300
C	5.12277900	-4.01177200	2.32820000	C	8.42206400	-2.38778700	-3.52236100

C	7.48382100	-3.15108700	-2.80406900	H	2.20424500	-3.11543700	-3.82698900
C	7.47164400	-3.17273900	2.79873700	H	2.19246400	-3.17963600	3.82206300
C	8.40686100	-2.41533600	3.52737800	H	3.75627800	-2.53652100	5.56189300
C	9.57310100	-1.95595300	2.91277500	H	6.17423700	-2.55991600	5.22069500
C	9.83086600	-2.14581900	1.54569300	H	-10.69681400	-1.65870100	1.10754600
H	-1.20410700	-4.84338000	3.32957900	H	-10.27441800	-1.36668600	3.49716500
H	1.20406000	-4.84337900	3.32958100	H	-8.21486500	-2.13805300	4.55933000
H	1.20341700	-4.78181500	-3.36892100	H	-8.23344500	-2.09922600	-4.55182500
H	-1.20346200	-4.78181400	-3.36892100	H	-10.29403500	-1.34694500	-3.47848000
H	-6.17427400	-2.55989600	5.22069400	H	-10.70781000	-1.66035800	-1.08983700
H	-3.75631500	-2.53651500	5.56189300	H	10.70778700	-1.66041800	-1.08983200
H	-2.19250500	-3.17963400	3.82206300	H	10.29401500	-1.34700200	-3.47847400
H	-2.20428200	-3.11543300	-3.82698800	H	8.23342000	-2.09926700	-4.55182100
H	-3.77172100	-2.44328600	-5.55273500	H	8.21483600	-2.13809300	4.55933300
H	-6.18920400	-2.48565300	-5.21140500	H	10.27439600	-1.36674400	3.49716900
H	6.18917200	-2.48565700	-5.21139200	H	10.69679000	-1.65876300	1.10755100
H	3.77169200	-2.44327900	-5.55272500				

8. References

(1) Still, W. C., Kahn, M. & Mitra, A. Rapid chromatographic technique for preparative separations with moderate resolution. *J. Org. Chem.* **43**, 2923–2925 (1978).

(2) Merz, J., et al., Pyrene molecular orbital shuffle-controlling excited state and redox properties by changing the nature of the frontier orbitals. *Chem. Eur. J.* **23**, 13164–13180 (2017).

(3) Ito, S., Tokimaru, Y. & Nozaki, K. Benzene-fused azacorannulene bearing an internal nitrogen atom. *Angew. Chem. Int. Ed.* **54**, 7256–7260 (2015).

(4) Sheldrick, G. M. University of Göttingen: Göttingen, Germany, 2014.

(5) Frisch, M. J. et al. *Gaussian 16, Revision A.03*, Gaussian, Inc., Wallingford CT, 2016.

(6) (a) Becke, A. D. *J. Chem. Phys.* **98**, 5648–5652 (1993). (b) Lee, C., Yang, W. & Parr, R. G. *Phys. Rev. B* **37**, 785–789 (1988).

(7) (a) Hehre, W. J. & Ditchfield, R.; Pople, J. A. *J. Chem. Phys.* **56**, 2257–2261 (1972). (b) Ditchfield, R., Hehre, W. J. & Pople, J. A. *J. Chem. Phys.* **54**, 724–728 (1971).
